# Quantification of free ligand conformational preferences by NMR and their relationship to the bioactive conformation^☆^

**DOI:** 10.1016/j.bmc.2013.06.056

**Published:** 2013-09-01

**Authors:** Charles D. Blundell, Martin J. Packer, Andrew Almond

**Affiliations:** a C4X Discovery Ltd, Unit 310 Ducie House, Ducie Street, Manchester M1 2JW, UK; b AstraZeneca, Alderley Park, Cheshire SK10 4TG, UK; c Manchester Institute of Biotechnology and Faculty of Life Sciences, University of Manchester, 131 Princess Street, Manchester M1 7DN, UK

**Keywords:** NMR, nuclear magnetic resonance, CSD, Cambridge structural database, PDB, protein data bank, NOE, nuclear Overhauser enhancement, Bioactive conformation, Solution conformation, NMR, Streptomycin, Aminoglycoside, Antibiotic, 30S ribosomal subunit, Ligand binding, Pharmacophore, Preorganization, Virtual screening

## Abstract

Accurate unbound solution 3D-structures of ligands provide unique opportunities for medicinal chemistry and, in particular, a context to understand binding thermodynamics and kinetics. Previous methods of deriving these 3D-structures have had neither the accuracy nor resolution needed for drug design and have not yet realized their potential. Here, we describe and apply a NMR methodology to the aminoglycoside streptomycin that can accurately quantify accessible 3D-space and rank the occupancy of observed conformers to a resolution that enables medicinal chemistry understanding and design. Importantly, it is based upon conventional small molecule NMR techniques and can be performed in physiologically-relevant solvents. The methodology uses multiple datasets, an order of magnitude more experimental data than previous NMR approaches and a dynamic model during refinement, is independent of computational chemistry and avoids the problem of virtual conformations. The refined set of solution 3D-shapes for streptomycin can be grouped into two major families, of which the most populated is almost identical to the 30S ribosomal subunit bioactive shape. We therefore propose that accurate unbound ligand solution conformations may, in some cases, provide a subsidiary route to bioactive shape without crystallography. This experimental technique opens up new opportunities for drug design and more so when complemented with protein co-crystal structures, SAR data and pharmacophore modeling.

## Graphical abstract

**Figure:**
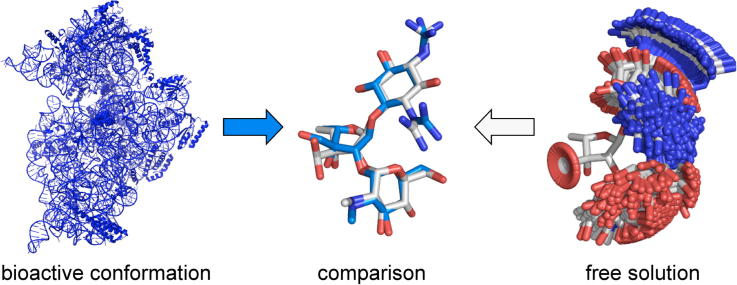

## 1 Introduction

Small molecules with high affinity for their targets bind using a particular 3D-shape and interaction network. This 3D-interaction map, or pharmacophore, can be used as a blueprint to enable the efficient optimization and design of molecules during the drug discovery process. Molecules designed to prefer the 3D-structure adopted in the bound state trend to higher association constants due to their conformational pre-organization.^1^ Documented successes of such conformational design strategies include the inhibition of HIV protease^2^ and BACE1.^3^ Similarly, many natural products and endogenous ligands take advantage of this conformational pre-organisation phenomenon by presenting a restricted group of conformations to a binding partner, retinol being a good example.^4^ Indeed, a significant number of natural products are based on rigid templates and frameworks that have limited conformational variability, e.g., alkaloids and steroids.^5^ In energetic terms, this conformational pre-organization lowers the conformational entropy penalty for transition from the ligand’s unbound set of conformations to its bioactive conformation(s).^6^ Therefore, as an important component of the observed binding affinity, a molecular understanding of the conformational changes upon receptor binding that a ligand undergoes could be harnessed to rationalize structure-activity relationships and design ligands with better affinity.^7^ Furthermore, when a ligand’s bioactive conformation cannot be directly measured (e.g., due to difficulties in crystallizing the target), the unbound conformation(s) of a high-affinity ligand can provide an alternative route to its bioactive conformation precisely because it is likely to be pre-organized towards the bioactive conformation to some extent, and this principle can enable ligand-based approaches to drug discovery and optimization.^8^

Solution NMR is the method of choice for measuring the unbound shapes of ligands because conformational restraints for use in 3D-molecular refinement can be directly measured from the ligand in its near-natural state. Conformational restraints typically used are spin relaxation data (e.g., NOESY, ROESY), scalar couplings^9^ and residual dipolar couplings.^10^ The most widely-employed 3D-refinement approach^11–13^ attempts to fit the restraints to a single rigid conformation, but the presence of multiple rapidly interchanging conformations often leads this process to produce virtual conformations that do not correspond to actual conformations ever adopted by the molecule.^14^ Other approaches try to address this problem by introducing ligand conformational flexibility with computational modeling methods.^15–19^ These have had mixed success because the methods variously rely upon accurately modeling complex electronic molecular orbital hybridization and strong interactions with solvent, neither of which are straightforward. For example, the method NAMFIS could not be applied to a polar molecule because current force fields did not uniformly represent its conformational profile.^20^ Yet other approaches attempt to reconstruct an analytical probability distribution for rotation about torsional angles from NMR data.^21^ These have proven unpopular because they are very complicated and have only been reduced to practice for an individual bond and are therefore of little practical value for conformational design.

**Figure 1:**
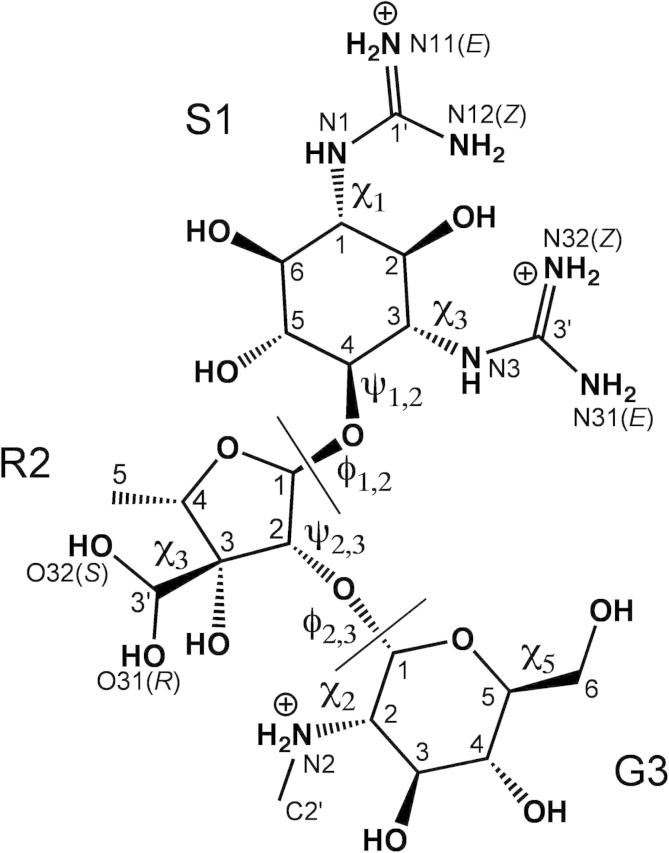
Chemical structure of streptomycin in aqueous solution. Streptomycin comprises streptidine (S1), streptose (R2, a ribose derivative) and glucosamine (G3) residues. Heavy atom designations within each residue are shown; prochiral atoms are indicated (*pro-S*, *pro-R*, *pro-E*, *pro-Z*). Glycosidic linkage and exocyclic torsion angles are marked and were defined as follows: *ϕ*_1,2_ = R2 O4–R2 C1–S1 O4–S1 C4; *ψ*_1,2_ = R2 C1–S1 O4–S1 C4–S1 C3; *ϕ*_2,3_ = G3 O5–G3 C1–R2 O2–R2 C2; *ψ*_2,3_ = G3 C1–R2 O2–R2 C2–R2 C1; *χ*_1(S1)_ = C6–C1–N1–C1′; *χ*_3(S1)_ = C2–C3–N3–C3’; *χ*_3(R2)_ = C2–C3–C3’–O31; *χ*_2(G3)_ = C1–C2–N2–C2’; *χ*_5(G3)_ = C4–C5–C6–O6.

**Figure 2:**
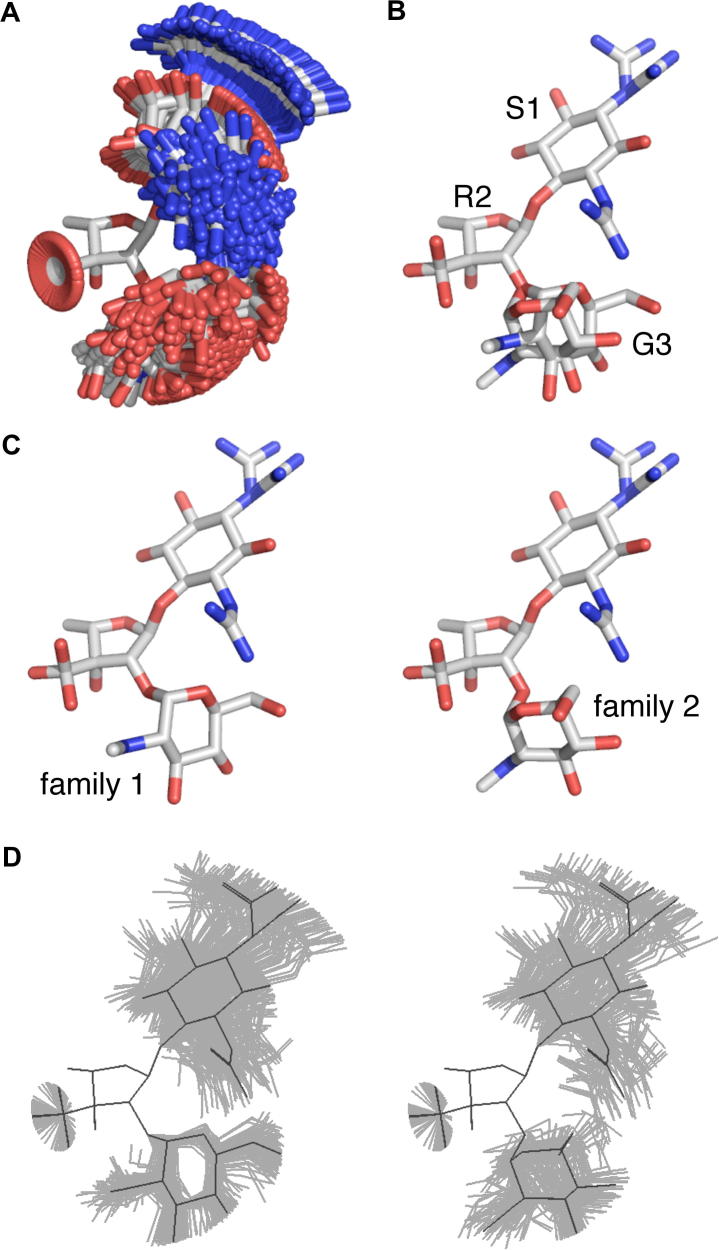
The dynamic 3D-structure of streptomycin in aqueous solution is remarkably well-defined in spite of the presence of multiple rotatable bonds (see Fig. 1). Each rotatable bond in streptomycin librates around one or more preferred macrostates (refer to Table 2), which can be represented by a uniformly weighted ensemble of 3D-structures whose distribution collectively accounts for the observed experimental data (A; the set of conformational microstates). This ensemble representation can be meaningfully reduced to a view where each rotatable bond is shown at the average position of each and every macrostate adopted (B). The bimodal behavior of the F2–G3 glycosidic linkage (*ϕ*_2,3_ and *ψ*_2,3_) gives rise to two principal conformational families (C, D; left vs right, Family 1 vs Family 2), between which the overall shape of streptomycin is significantly different. These two families are shown in the set of macrostates (C) and transparent overlay views (D). The overlay captures the relationship between the extent of libration (grey; microstates) about each macrostate (black) for each family. The position of each residue is labeled; all molecules have been overlaid on the heavy atoms of the R2 furanose ring. Carbon atoms are shown in white, nitrogen in blue and oxygen in red; hydrogen atoms have been omitted for clarity.

Here, we present and demonstrate a new NMR methodology that is able to accurately quantify the conformations of unbound ligands in solution (i.e., to effectively reproduce the Boltzmann distribution) to the resolution needed for drug design. The technique uses multiple datasets and a dynamic model during refinement of the ligand, does not employ computational chemistry techniques to mimic molecular flexibility and avoids the problem of virtual conformations. We applied the method to the aminoglycoside streptomycin (Fig. 1) and found that the resultant dynamic 3D-structure was best described by two families of conformations (Fig. 2). The dominant conformational family has an identical 3D-structure to the observed bioactive conformation of streptomycin when bound to the 30S ribosomal subunit, consistent with the expectation that streptomycin is conformationally pre-organized for binding to its target.

Accurate unbound ligand 3D-structures as determined here can be used to provide detail on the contribution of ligand conformational reorganization energies on binding and can, moreover, be used in lieu of co-crystal data as predictors of the bioactive conformation, particularly in the case of high affinity ligands. These proxies for the bioactive conformation could be used throughout hit identification and lead optimization processes both to discover new chemistry starting points and to drive the optimization of affinity and selectivity of compounds using conformational design.

## 2 Materials and methods

### 2.1 NMR sample preparation

Streptomycin was purchased from Sigma–Aldrich (sulfate salt, product number S1937, CAS number [3810-74-0]). Samples for data acquisition were prepared at a range of concentrations (0.1–50 mM) in solutions of 100% D_2_O or 90%:10% (v/v) H_2_O/D_2_O, pH^∗^ 6.0, and contained 1 mM *d*_6_-DSS as an internal reference.

### 2.2 Chemical shift assignment

All ^1^H, ^15^N and ^13^C chemical shifts and pertinent coupling constants of streptomycin in aqueous solution at pH 6.0 were assigned using standard 1D and 2D-NMR experiments at 600 MHz (refer to Fig. 1 for nomenclature and Supplementary Tables S1–S8 for assignment data). Chemical shifts and coupling constants indicate that the aldehyde group of streptomycin at R2 C3′ was predominantly hydrated (96 ± 1%; 5 °C in H_2_O) in aqueous solution, giving the *gem*-diol form as shown in Figure 1. Coupling constants, chemical shifts and the absence of any specific inter-residue correlations in ^13^C- and ^15^N-HMBC spectra preclude the proposed aldehyde-cyclized forms of streptomycin in aqueous solution.^22–24^

Chemical shifts and conformation-dependent ^3^*J*_HH_ coupling constants were measured at several solute concentrations (0.1–50 mM streptomycin) and at different temperatures (5 –35 °C). Since the differences in ^1^H chemical shifts between these conditions are small (<0.1 ppm), all temperature-induced perturbations occur linearly (see Supplementary Figure S1 for regression plots), and no ^3^*J*_HH_ coupling constant changes by more than 0.2 Hz, it was concluded that streptomycin does not significantly self-associate in aqueous solution and its dynamic 3D-structure is insensitive to changes in temperature in the physiological range. The glucosamine secondary amine (G3 N2) was fully protonated at pH 6.0 (distinct HN21 and HN22 protons were observed in COSY experiments) and therefore (including the two guanidinium groups) streptomycin carries a +3 charge under physiological conditions. Pro-chiral stereo-assignments were determined during structure calculations (see below).

### 2.3 Structural restraint measurement

#### 2.3.1 Conformation-dependent scalar couplings

Three-bond homonuclear coupling constants (^3^*J*_HH_) used for structure determination were measured from ^1^H-1D spectra recorded at 900 MHz (16 384 complex points, dwell-time 92.5 μs) on both D_2_O and H_2_O samples. Scalar couplings were measured by a resonance line-fitting algorithm that moved a set of modeled scalar couplings until a fit was obtained with the experimental multiplet pattern;^25^ the Lorentzian line width used by the algorithm was also obtained as part of the fit. The resultant tabulated data are presented in Supplementary Table S7. For each coupling constant a suitable Karplus relation (typically an incarnation of the Altona–Hasnoot equation^26^) for the torsion in question was associated with the raw measurement to produce a structural restraint. Even though the couplings were measured with an error of 0.1–0.4 Hz, because the Karplus equations only have a predictive accuracy of ∼1 Hz,^26^ this value was used as the error for the restraint. The derived structural restraints are presented in Supplementary Table S9.

#### 2.3.2 Nuclear overhauser enhancements (NOEs)

A 2D-NOESY spectrum with pre-saturation for water suppression was acquired on a 50 mM 100% D_2_O sample at 900 MHz and 5 °C with a mixing time of 700 ms (acquisition dimension—2048 complex points, dwell-time 92.5 μs; indirect dimension—256 complex points, dwell-time 109.9 μs). A second 2D-NOESY spectrum with WATERGATE water suppression^27^ was recorded using a 50 mM 90%:10% (v/v) H_2_O/D_2_O sample at 600 MHz and 5 °C with a mixing time of 600 ms (acquisition dimension—4096 complex points, dwell-time 138.9 μs; indirect dimension—128 complex points, dwell-time 138.9 μs). Detailed experimental information was extracted from these 2D-NOESY experiments by measuring the absolute values of cross-peak heights directly from the NMR spectra. All possible cross-peak positions were measured, including overlapping peaks and those that were found to be unambiguously zero, but excluding positions that could not be measured due to NMR artifacts. Although the majority of hydroxyl protons in streptomycin were observed and assigned at 5 °C, they remained in such fast exchange that it was not possible to measure structural restraints to them and they are therefore excluded from the 3D-structure described here. Due to the resolution of the spectrum in the acquisition dimension, the fine structure for each cross-peak was visible. Each component of the fine structure was base-lined and rescaled by multiplication by suitable scaling factors to allow the correct equivalent peak-height value for one mole abundance of protons to be calculated. These scaling factors were readily calculated for every fine structure component from the coupling constants already measured for each proton. For example, each component in a doublet had a scaling factor of 2, whereas those in a triplet were 4, 2 and 4. The mean peak-height value calculated from the fine structure components for each cross-peak was taken as the peak-height value for that cross-peak. In this way, the true relative peak-height for each cross-peak in the 2D-NOESY spectrum was measured while compensating for the problem of the different line-shapes of each proton making peaks appear at first inspection more or less intense than they really were. The error on each 2D-NOESY cross-peak was set at 40% of the measured height (to take into account both measurement and prediction errors). When there was no signal intensity above the noise of the spectrum at the cross-peak position where a correlation may have been possible, a ‘no-NOE’ was assigned. Such no-NOEs were given a true peak-height of zero and their standard errors were set to a third of the value of the maximum peak-height calculated from the height of the noise at the chemical-shift coordinates and the proton’s scaling factors (i.e., treating the noise as though it could have been the top of a peak from the most intense multiplet component, within 3 standard deviations). All experimental restraints derived from the NOESY spectra were therefore given both a height value equivalent for one mole of protons (either a value for NOEs or zero for no-NOEs) and an associated measurement error. To avoid the problem of converting no-NOEs into meaningless distance restraints for structure calculations, these peak-heights were worked with throughout (see below). The experimental restraints derived from the 2D-NOESY spectra in D_2_O and H_2_O are shown in Supplementary Tables S10 and S11, respectively.

#### 2.3.3 Residual dipolar couplings

Strained gels were prepared using 4.5% and 6% (w/v) polyacrylamide as described previously^28^ and soaked in a solution of 50 mM streptomycin in 100 mM D2O pH^∗^ 6.0, before insertion into a suitably prepared 5 mm NMR tube. Coupling constants (^1^*J*_CH_) were measured directly from the acquisition dimension (600 MHz, 4096 complex points, 1.13 s acquisition) of ^13^C-HSQC spectra recorded without broadband ^13^C-decoupling during acquisition. Residual dipolar couplings were calculated from the difference in observed H–C splitting between the two sets of spectra recorded in the free solution and aligned phase. The error in each measurement was estimated from the respective 2D NMR spectra by taking measurements that were clearly greater or smaller than the observed H–C splittings; errors were 1–2 Hz. The derived experimental restraints are tabulated in Supplementary Table S12.

#### 2.3.4 3D-Structure calculations

An initial 3D-structure for streptomycin was built with fixed bond lengths and angles (based on geometrical constraints rather than by minimizing a computational chemistry energy function), which when compared with the Cambridge Structural Database (CSD) had Mogul *Z*-scores less than 3 (see below). Ensembles for streptomycin were constructed by keeping all bonds and angles fixed and rotating bonds according to a specific algorithm. Each rotatable bond was assigned a set of conformer modes, or in statistical thermodynamic terms, macrostates (*Σ*); each macrostate comprising a mean torsional angle (*μ*), a spread (*σ*) and a probability (*π*). The number of macrostates associated with a bond could be varied to allow it to adopt several principal conformations, i.e., uni-modal, bimodal or tri-modal. For example, consider a tri-modal bond (e.g., sp^3^–sp^3^) with three macrostates $\Sigma_{1}[\pi_{1}\mu_{1}\sigma_{1}]$, $\Sigma_{2}[\pi_{2}\mu_{2}\sigma_{2}]$, $\Sigma_{3}[\pi_{3}\mu_{3}\sigma_{3}]$. Clearly, *π*_1_ + *π*_2_ + *π*_3_ = 1 and thus the macrostate can be selected using a random number, after which an angle can be randomly selected for the chosen macrostate using the mean and spread and standard stochastic computational techniques^29^ to generate individual bond microstates. Rules for selection of conformer macrostates^30^ are devised for every bond (which we term a dynamic model, see below) and microstates are selected at all rotatable bonds in the molecule, from which an ensemble of microstates for the whole molecule is created by repeatedly performing this process. For streptomycin, the size of the generated ensemble (total number of microstates) was fixed at 300, which was found to satisfactorily cover the molecular conformational space.

The ensemble of microstates, which as a group could collectively represent the conformations actually populated in aqueous solution, was used to make predictions of experimental data via an appropriate theory. In the case of the NOESY experiment, inter-proton distances (*r*_ij_) were averaged over the ensemble according to 〈*r*_ij_^−6^〉 and the resultant matrix converted to a relaxation rate matrix by multiplication by spectral densities estimated from an overall tumbling correlation time (*τ*_c_). The relaxation matrix first-order rate equation was solved using diagonalization methods described previously.^31^ The predictions of cross-peak heights produced by this method were used directly in structure calculations without further processing. The predicted cross-peak heights were compared against the experimentally measured cross-peak heights by means of a chi-square least-squares function (see below). The value of *τ*_c_ was optimized during structure calculations to ensure the best possible agreement between predictions and experimental data. It should not be considered that only the NOEs (or no-NOEs) between protons separated by rotatable bonds contain useful conformational information because the observed intensities of those NOEs between protons related by fixed geometries within the molecule (e.g., intra-ring) should also be correctly predicted in the final 3D-structure; in short, all cross-peak positions contain conformational information and all should be correctly predicted by the final 3D-structure. To predict scalar couplings, standard Karplus equations were employed^26,32^ and the calculated values (*J*) were averaged over the ensemble 〈*J*〉 and used in structure calculations without further modification. Residual dipolar couplings were calculated from the ensemble using a method based on molecular shape, described previously^33^, in combination with a generalized alignment tensor.^34^ The dependence on angle is highly non-linear and thus an extra error correction was applied to the experimental error dependent upon the angle (see Supplementary data).

A chi-square least-squares measure (*χ*^2^) was used to determine the goodness of fit between the experimental data (*x*_exp_) and the theoretical predictions (*x*_pred_), which is the sum of the square distances between prediction and experiment, divided by the square of the estimated error (${∊}_{\text{exp}}^{2}$) on each experimental measurement. For the ensemble the fitness function *χ*^2^ was calculated from the predictions and the measured experimental data. A computer optimization was conducted to minimize *χ*^2^ by varying the probabilities, means and spreads for each state, until the best fit was found using 10 000 steps of the Metropolis Monte–Carlo method.^35^ This optimization was typically run 96 times (with random starting configurations) and the 10% of runs with the lowest *χ*^2^ were compared to ensure that the optimization had converged to the same set of conformations.

There are nine rotatable bonds in streptomycin (Fig. 1) that can adopt one or more conformations (four in the glycosidic linkages and five exocyclic groups). As in other NMR structure-determination processes, iterative rounds of calculations were performed in which the types of bond macrostates (bi-modal, tri-modal) applied to each bond within streptomycin was varied. The ability of each of these dynamic models to predict the observed experimental data was scored. The models were altered until the best possible fit found (in particular that all experimental data were correctly predicted) and that the calculations had converged to a consistent set of conformations. Initial rounds of structure calculations used as many unambiguous structural restraints as possible and were performed with dynamic models that allowed each rotatable bond to adopt three different mean angles of any value, each with attendant floating libration amplitudes (i.e., tri-modal behaviors of unspecified geometries). When the experimental data were seen to define consistently the behavior of bonds as having only one or two mean angles (i.e., uni-modal or bi-modal), the dynamic model was reduced accordingly. As the structure became more defined, ambiguous restraints could be assigned and were included more specifically. Also, as data were included they could eventually be used to determine pro-chiral stereo-assignments by systematically performing calculations on the two NMR-degenerate combinations at each center and selecting the combinations with the lowest *χ*^2^-score. Bonds that were seen to settle on classic rotamer angles (i.e., 60°, 180°, 300°) had their macrostates fixed in later rounds of structure calculations. Ring pucker was handled by changing the dihedral angles in the ring to canonical pucker states. In this way, all possible puckers could be tested against the experimental data individually and in combination with other puckers by linking the sets of dihedral angles to higher modes (bi-modal, tri-modal).

Once no change to the dynamic model could reduce the total *χ*^2^-score, then it was declared as the final dynamic model (see Supplementary Tables S9-S12 for final χ2-scores for each restraint). For streptomycin no other fundamentally different model could be found that could achieve this (i.e., the solution was singular). Furthermore, assuming that the restraints are spread evenly throughout the molecule (as they were in streptomycin; see Supplementary Table S13), then the ensemble of microstates produced by this final model was able to predict all experimental data simultaneously and hence this ensemble must be a good approximation to the Boltzmann distributed dynamic conformations of streptomycin in solution. Two sets of structures were output at the end for use in medicinal chemistry or otherwise (see Supplementary data).

The first set of co-ordinates was an ensemble with 250 individual conformational microstates that are generated from the full dynamic model. Individual conformations that brought atoms closer than allowable according to the CSD (e.g., S1 O5 and R2 O4 closer than 2.4 Å) were trimmed from the final output conformational ensemble and replaced by another acceptable conformation to retain a total of 250. This modification allowed the total *χ*^2^ score to be decreased slightly, but the mean values for each mode were essentially unperturbed. Modifications of this type were kept to an absolute minimum in order to minimize counter-productive artificial distortions to the set of 3D-structures.^36^

The second set of co-ordinates was a more degenerate set of conformational macrostates, comprising only the mean states (without libration), which is effective in detailing the major conformational states and their populations (calculated from the probabilities of the underlying states). Trivial bond rotations not affecting the overall conformation of the molecule, e.g., methyl rotation were effectively removed from this set by grouping them together.

### 2.4 Comparison of bond lengths and angles with the Cambridge structural database

An analysis of the correctness of the 3D-structures (particularly bond lengths and bond angles) used in this work was conducted by comparing them with data from the Cambridge structural database (CSD)^37^ via the Mogul software package.^38^ Where bond lengths or bond angles deviated significantly from those observed previously, it is most likely that there is an error in model building that should be addressed.^39^ The threshold for rejecting a 3D-structure was a Mogul *Z*-score of more than 3. All input structures, calculated conformational microstates and macrostates for streptomycin were confirmed to have Mogul *Z*-scores less than 3, indicating that all values fall within the set of more than 99% of previous observations.

### 2.5 Computational modeling of streptomycin

Torsional profiles were constructed for streptomycin by scanning the two *ϕ*/*ψ*-angle pairs at intervals of 10° in a grid-like fashion. Each conformer was optimized for every other conformational variable except the *ϕ*/*ψ* angles under consideration using the Macromodel package,^40^ with the OPLS2005 force-field,^41^ an implicit GB/SA model for water.^42^ Following optimization the final energy was recorded to allow construction of a contour plot and the coordinates stored for later analysis, e.g., of ring puckers.

## 3 Results

### 3.1 Structural restraints

In total, 309 experimental structural restraints were used to define the dynamic 3D-structure of streptomycin (Table 1; see Supplementary Tables S9-S12 for details), comprising nuclear Overhauser enhancements (NOEs), scalar couplings and residual dipolar couplings. The values of the *χ*^2^ per restraint score between different datasets are similar, indicating that experimental data was uniformly weighted in the final structure. The majority of NOE restraints were measured from a sample in D_2_O and the NOEs in this dataset were well predicted (*χ*^2^ per restraint = 0.8). NOE restraints measured from a sample in H_2_O to the exchangeable guanidinium hydrogen atoms are the most poorly predicted (*χ*^2^ per restraint = 1.5), probably because the differential exchange rates of the guanidinium hydrogen atoms with respect to each other made accurate measurement of relative cross-peak heights difficult. In total, 116 NOE and 87 no-NOE inter-residue restraints were measured (Table 2), allowing the conformational behavior of the glycosidic linkages in particular to be thoroughly interrogated and characterized. The residual dipolar coupling measurements, which are particularly sensitive to the global shape of the molecule, were very well predicted by the dynamic 3D-structure (*χ*^2^ per restraint <0.5) indicating moreover that the overall shape of the molecule was correct. The structural restraints were evenly distributed throughout the molecule and therefore all parts of the molecule were comparably defined (Supplementary Table S13). Furthermore, individual datasets (e.g., the residual dipolar couplings or NOEs) could each be used in the absence of the others to predict essentially the same conformational ensemble independently; albeit to a lower accuracy (this also indicates that the presence of the gel has not perturbed the shape of streptomycin and that therefore the residual dipolar couplings are reliable). When all the data was used simultaneously the overall accuracy of the structure improved substantially.

### 3.2 A more accurate dynamic view of streptomycin

As shown in Figure 1, there are nine rotatable bonds in streptomycin (four in the glycosidic linkages and five exocyclic groups) and three saturated rings that needed to be characterized to describe fully the solution structure of streptomycin. The dynamic structure should be interpretable in statistical thermodynamic terms if it is to be useful as a predictive medicinal chemistry aid. The most straightforward representation of the dynamic structure (as described in Table 3) is a set of 3D-structures that reflect uniform sampling from the Boltzmann distribution (i.e., as used in the structure determination process itself); this set will be referred to as the set of conformational microstates (in this case comprising 250 3D-structures) and shown in Figure 2A. Another helpful simplification of the actual dynamic solution behavior is the set of the major conformational states at each bond (e.g., a bond with two major states will be bi-modal), which are referred to as macrostates. In this case, streptomycin as a whole was found to have 12 conformational macrostates (shown in Fig. 2B). The macrostates represent major conformations around which the microstates librate.

Following conformational refinement it was found that the experimental data had constrained the solution structure to a remarkably limited and well-defined set of conformations (Fig. 2), principally due to the behavior identified at the two glycosidic linkages and the R2 ribose ring pucker. The R2 ribose ring conformation was very clearly defined as being uni-modal and ^4^*E* (to the nearest twist/envelope conformation, i.e., 144 ± 9°). The large vicinal ^3^*J*_HH_ coupling constants, sterics and residual dipolar couplings defined the S1 streptidine and G3 glucosamine rings as ^1^C_4_ and ^4^C_1_ chairs, respectively. The S1–R2 linkage adopts a single mean conformation (*ϕ*_1,2_ = −75 ± 7°, *ψ*_1,2_ = −134 ± 17°; see Table 3), about which it librates (ϕ_1,2_ = 16 ± 7°, *ψ*_1,2_ = 22 ± 6°). The R2–G3 linkage adopts a bi-modal behavior, librating about two distinct conformations (primary, 62 ± 16%, *ϕ*_2,3_ = −103 ± 6°, *ψ*_2,3_ = −81 ± 9°; secondary, 38 ± 16%, *ϕ*_2,3_ = 103 ± 8°, *ψ*_2,3_ = 173 ± 14°). The more populated primary mode has a restricted range of movement with small librational amplitudes (*ϕ*_2,3_ = 6 ± 1°, *ψ*_2,3_ = 12 ± 7°), while the secondary mode has larger librational amplitudes (*ϕ*_2,3_ = 10 ± 5°, *ψ*_2,3_ = 25 ± 11°) that are similar to those of the S1–R2 linkage. The R2 C3’ gem-diol group (*χ*_3(R2)_) occupies all three classic rotamer positions, with the experimental data distinctly disfavoring the 60° conformation during refinement (only 4 ± 3% of the total). The G3 secondary amine (*χ*_2(G3)_) prefers a single conformation independent of the conformation of the R2–G3 linkage. The S1 C1 guanidinium group (*χ*_1(S1)_) is best fit by a bimodal behavior (average angles symmetrical about the S1 H1 hydrogen) that is independent of any other motions in the molecule. In contrast, the S1 C3 guanidinium group (*χ*_3(S1)_) distinctly prefers a uni-modal behavior. It is suggested that this is caused by steric interactions with the G3 C6 hydroxymethyl group. Supporting this, the chemical shifts of the protons of the guanidinium group at N31 are distinctly resolved from N32, which has the ^15^N and ^1^H chemical shifts characteristic of exposure to bulk solvent. Since the G3 C6 hydroxymethyl group is in very close contact with this guanidinium group (Fig. 2B and C) in both conformations of the R2–G3 linkage, it might therefore be expected that the hydroxymethyl conformation (*χ*_5(G6)_) would depend on the R2–G3 linkage. Indeed, the best fit to the experimental data was observed when the *χ*_5(G5)_ bond adopted slightly different mean angles for each R2–G3 linkage conformation. In both R2–G3 linkage conformations the best fit model for the *χ*_5(G5)_ bond was a uni-modal behavior with an average angle close to the ideal rotamer position of 300°.

### 3.3 Conformational families

All the measured experimental data was satisfactorily described by a dynamic 3D-structural model in which streptomycin librates about just 12 macrostates (Fig. 2B; two conformations for the R2–G3 linkage, permuted by three for the R2 C3′ *gem*-diol group *χ*_3(R2)_ and a further two for the S1 C1 guanidinium group χ_1(S1)_), whose overall occupancies can be readily calculated from Table 3.

Since only the two distinct conformations of the R2–G3 linkage significantly affect the overall shape of the molecule, it is helpful to cluster the 12 conformational macrostates into two distinct families (Fig. 2C) dependent upon this linkage. From Table 3, the populations of Family 1 and Family 2 are measured to be 62% and 38%, respectively, (with an error of 16% either way on this split). The extent of libration within each family relative to the mean position for each conformation is shown in Figure 2D. It is important to note that the spread shown in Figure 2D is not indicative of the error in the structure determination process, but represents the true dynamic range of motion of the molecule in solution.

### 3.4 Inferred intramolecular interactions

Across the S1–R2 linkage, the S1 O2 hydroxyl group is positioned very favorably to donate a hydrogen bond to the R2 O5 ether oxygen in the set of conformational macrostates (S1 O2–R2 O5 distance = 2.7 Å; see Supplementary Fig. S5). Consistent with this, the S1 HO2 proton is visible in NMR spectra up to 25 °C (fully 10 °C higher than any other hydroxyl proton in streptomycin), indicating that it is markedly less susceptible to exchange with bulk solvent for some reason. However, its large temperature coefficient (−11.9 ppb/K) indicates that if this hydrogen bond forms, it is probably only partially populated and highly transitory, reflective of the nature of this type of hydrogen bond.

In the set of macrostates of the dominant mode of the R2–G3 linkage (i.e., Family 1), the G3 HN21 hydrogen is very well positioned to form a transitory hydrogen bond with either *gem*-diol oxygen (R2 O31 & O32) as the *χ*_3(R2)_ bond rotates having near-optimal distances of 2.7 Å (N21–O31/32) and 1.7 Å (HN21–O31/32) and an angle (N21–HN21…O31/32) of 158°.^43^ It is noted that the 60° conformation for the *gem*-diol group does not allow this hydrogen bond to form because it positions the R2 H31 proton towards the G3 HN21 proton. As assessed by the set of conformational microstates, there are also multiple potential transient hydrogen-bond interactions between the S1 C3 guanidinium group and the G3 residue (S1 HN311-G3 O6 & O5, S1 HN3-G3 O6 & O5) that could be reached through libration within the Family 1 set of conformations.

In the less populated mode of the R2–G3 linkage (i.e., Family 2), the G3 HN21 hydrogen is not close enough to form a transient hydrogen bond to either *gem*-diol oxygen. In the set of conformational macrostates (i.e., at the average linkage positions), G3 HN21 is also not close enough to the R2 O3 oxygen atom to make a transient hydrogen bond; however, the set of microstates show that it can be reached through libration. There are also some potential transient hydrogen-bond interactions between the S1 C3 guanidinium group and the G3 residue (S1 HN311-G3 O6 & O4) that could be reached through libration. The fewer (and likely less occupied) potential transient hydrogen bonds in Family 2 compared to Family 1 may underlie its lower abundance in aqueous solution.

## 4 Discussion

### 4.1 Comparison with the prior NMR solution structure

A prior combined NMR and molecular dynamics study utilized 14 experimentally-determined structural restraints (13 NOE, 1 ^3^*J*_HH_) in restrained molecular dynamics simulations to characterize the conformation of streptomycin in aqueous solution.^44^ This work concluded that streptomycin adopts two main conformations in solution (S1–R2 *ϕ*_1,2_ 55°, *ψ*_1,2_ 25° & *ϕ*_1,2_ 30°, *ψ*_1,2_ −50°; R2-G3 *ϕ*_2,3_ 55°, *ψ*_2,3_ 45° & *ϕ*_2,3_ 30°, *ψ*_2,3_ −60°), in which both linkages change conformation together in a concerted motion. In contrast, the dynamic 3D-structure determined in this work with 309 experimentally-determined structural restraints without restrained molecular dynamics simulations indicates that the linkages adopt quite different conformations (S1–R2 *ϕ*_1,2_ −75°, *ψ*_1,2_ −134°; R2–G3 *ϕ*_2,3_ −103°, *ψ*_2,3_ −81° & *ϕ*_2,3_ 103°, *ψ*_2,3_ 173°) and that their motions are not concerted. A third minor (<4%) linkage conformation was also concluded to be necessarily present in this earlier work in order to account for two particular NOEs (R2 H2-S1 H4 and R2 H4-G3 H4), however these NOEs fit the two-family dynamic 3D-structure determined here extremely well (*χ*^2^ scores of 0.56 and 1.06, respectively) without the presence of this third conformation in the ensemble.

The previous study of streptomycin concluded that the ribose ring was flexible, presenting two populations with puckering values of 100–175° (*E*_3_/^4^*T*_4_/^4^*E*/^4^*T*_O_) and 210–300° (^1^*E*/^1^*T*_2_/*E*_2_/^3^*T*_2_/^3^*E*/^3^*T*_4_), however the larger set of experimental data used here is only consistent with a single moderately rigid pucker with an angle of 144 ± 9° (^4^*E*). Using four structural restraints to the R2 H3′ proton in the restrained molecular dynamics simulations, the *gem*-diol group was previously determined as being in a single antiperiplanar conformation. However, based upon 35 structural restraints, this work concludes that a second major conformation is necessarily present at the *gem*-diol group to account for all the observed experimental data.

### 4.2 Comparison with protein/RNA-streptomycin complex structures

**Figure 3:**
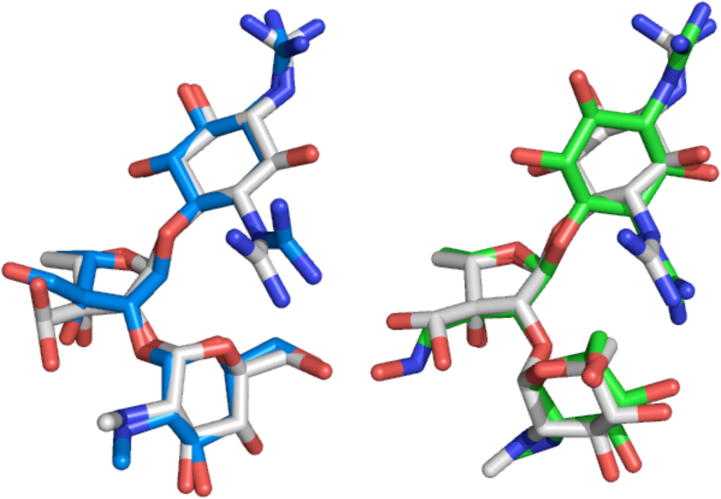
Overlays of the dynamic conformation of streptomycin in aqueous solution (carbon atoms in white) with conformations derived from X-ray crystallographic studies (carbon atoms in blue or green). (Left) The bioactive conformation of streptomycin (blue) from its co-complex with the 30S ribosome particle (PDB code 1FJG) is very similar to Family 1 conformational macrostates (the most populated) present in aqueous solution, demonstrating that the bioactive conformation of streptomycin is encoded within its preferred unbound solution conformations. (Right) The free crystal structure of streptomycin oxime shown in green^57^ corresponds closely to Family 2 conformational macrostates present in aqueous solution. As would have been predicted on steric grounds from the dynamic conformation of streptomycin (see Fig. 2A), derivatization to the oxime has apparently not significantly affected the conformational preferences of the F2–G3 glycosidic linkage. Molecules were overlaid on the heavy atoms of the rings from all 3 residues.

**Figure 4:**
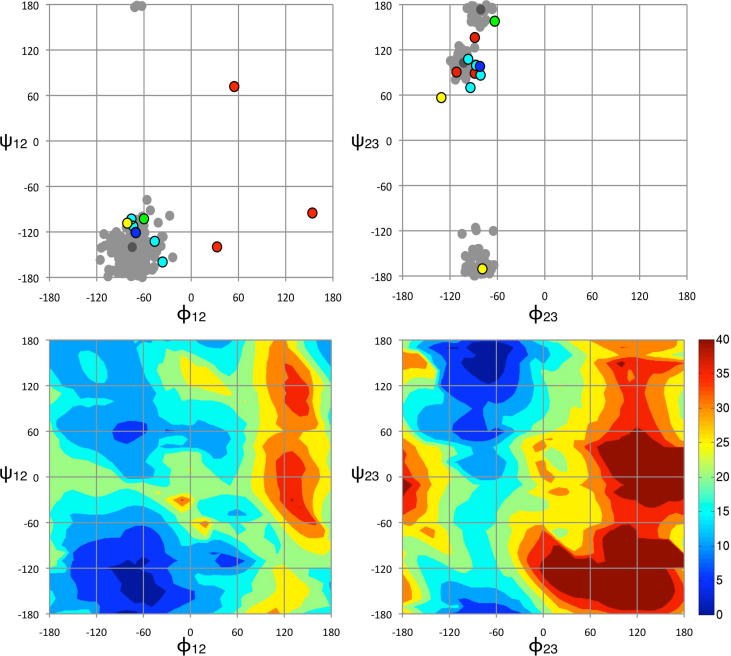
(Top) Comparison of the glycosidic linkage torsion angles for the aqueous solution dynamic 3D-structure of streptomycin determined in this work (grey) with published crystal conformations (colors). The extent of libration at each linkage is taken from the set of conformational microstates (light grey) while the average positions are taken from the set of conformational macrostates (dark grey). The S1–R2 linkage (left) adopts a uni-modal conformational distribution in solution, whereas the R2–G3 linkage (right) adopts a bi-modal distribution. The highest resolution bioactive conformation (i.e., when streptomycin is bound to the ribosome, PDB code 1FJG) is shown in blue, lower resolution ones in cyan (PDB codes 4DR3,5–7) and the free crystal structure^57^ is in green. The conformations of streptomycin bound to artificially-selected RNA aptamers (1NTA, 1NTB) are given in yellow (one conformation of the S1–R2 linkage lies directly below the blue bioactive conformation so is not visible) and the three conformations measured from the off-target low affinity co-complex with aminoglycoside-6-adenyl-transferase are given in red (3HAV). (Bottom) A computational chemistry grid-search performed at each of the glycosidic linkages of streptomycin. Energies (kcal/mol) are shown in false color (see key) and comparison with the top panel reveals that the dynamic solution 3D-structure of streptomycin determined here has found all low energy basins for the molecule solely on the basis of experimental data. Also, it is noted that aminoglycoside-6-adenyl-transferase (3HAV) conformations shown in the top panel in red lie within regions predicted computationally to have the highest energies.

The conformation of streptomycin bound to the bacterial 30S ribosomal subunit (its natural target), has been determined to 3.0 Å (PDB code 1FJG)^45,46^ and to ∼3.5 Å (PDB codes 4DR3 and 4DR5–7) in a range of *apo*- and tRNA-bound states. An overlay of the coordinates of the highest-resolution structure (PDB code 1FJG) with the most populated mode conformation in aqueous solution (i.e., the Family 1 linkage conformation) demonstrates that (within error of the crystal structure) the most populated conformational family in aqueous solution is the same as the bioactive conformation (Fig. 3). This can also be seen in Figure 4, where glycosidic angle torsions have been plotted for the structure determined here (in grey) and for the bioactive conformation (blue). Comparison of the lower resolution bioactive structures (cyan) also shows that streptomycin has the same conformation at both linkages, irrespective of the ribosomal state. Interestingly, the spread of these bioactive conformations is similar to the librational spread measured in solution (although this might be a consequence of the lower resolution). It is therefore difficult to ignore the obvious implication, namely that the unbound solution conformation of streptomycin has been selectively optimized to be as favorable as possible for interacting with its target; that is, the unbound solution conformation of streptomycin is pre-organized for optimal binding.

**Figure 5:**
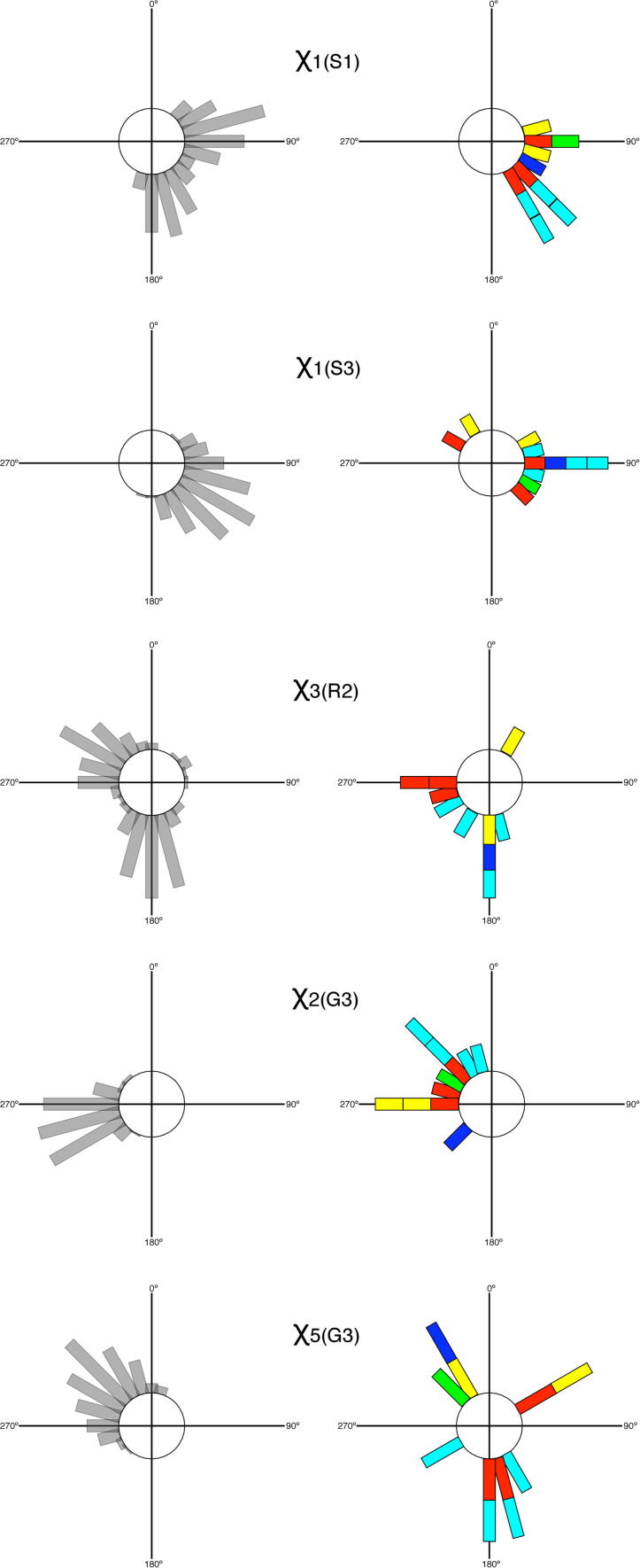
Circular histograms comparing the exocyclic torsion angles for the solution 4D-structure of streptomycin determined in this work (left, grey) with published crystal conformations (right, colors). Colors are as in Figure 4 (blue/cyan = bioactive; green = free crystal; yellow = RNA aptamers; red = off-target interaction). Conformations were binned at 15° intervals, centered on 0° at north and proceeding clockwise.

Considering the unbound and bound structures of streptomycin in more detail (Figs. 2 and 3), the highest-resolution crystallographically-derived bioactive conformation of the S1–R2 linkage is within 14° of the mean position (dark grey) adopted in solution (and, within error, is indistinguishable). Moreover, all the bioactive conformations lie within the ensemble of microstates naturally explored in solution (light grey). The bioactive R2–G3 linkage conformations are also very similar to the Family 1 linkage average position and lie within the ensemble of microstates this linkage explores in solution. The highest-resolution bioactive R2 ribose ring pucker conformation (165°; ∼^4^*T*_O_) is adjacent to that seen when unbound (144 ± 9°; ^4^*E*) (see Supplementary Fig. S6). All the exocyclic torsion angles of the highest resolution structure (and many of the lower resolution structures) are also found at either the mean position of solution macrostate conformations or within the unbound librational range (Fig. 5).

While most of the bond lengths, angles and torsions within the highest-resolution bound conformation of streptomycin are in good agreement with the existing CSD data (see Table 4), the conformation of the S1 C1 guanidinium group is particularly unusual (with a significant twist that breaks the planarity of the group). Even though this group forms distinct interactions with the ribosome, such a conformation has never been observed in solution and is so energetically unfavorable that it seems quite improbable. However, were this group to retain the flexibility observed when unbound upon binding (i.e., oscillation between two modes; see Figs. 2C and 5), the electron density map could be effectively accounted for; it is therefore proposed that the X-ray data is better accounted for by a structural model in which the flexibility observed in solution for this group is retained on binding.

The bound conformation of streptomycin is given in the free aldehyde form (Fig. 3), rather than the hydrated *gem*-diol form that predominates in aqueous solution (97%). This difference could be accounted for by proposing that only the aldehyde form is able to bind, that streptomycin dehydrates within the binding site or that there was insufficient resolution in the electron density map to observe the additional oxygen atom.

Two different crystal forms of the complex between streptomycin and 40-mer RNA aptamers have been reported.^47,48^ These aptamers were selected using dihydrostreptomycin coupled to sepharose and specific binding to the S1 C1 guanidine group of dihydrostreptomycin was achieved using bluensomycin for counter-selection. In spite of this selection environment being so different to free solution conditions, the two aptamer bound conformations remain remarkably similar to those seen in the unbound state at both linkage (Fig. 4) and exocyclic torsions (Fig. 5); indeed, the two crystal forms themselves are more different from each other than they are from the free dynamic-3D structure.

Streptomycin binds with low-affinity to aminoglycoside-2′′-phosphotransferase, and three different bound co-complex conformations were reported from a single crystal.^49^ Since streptomycin binds with low-affinity, it might be expected that the bound conformations differ significantly from those seen in the unbound state. Unfortunately, the bound conformations have a high number of unlikely bond lengths and angles (as assessed by Mogul^38^) and several stereocenters are incorrect. Furthermore, this crystal structure could not be independently validated by reproduction,^50^ which is highly suggestive of a problem with the original refinement. Given these problems, it is difficult to make conclusive comparisons with the free solution structure. Nevertheless, the conformations of the R2–G3 glycosidic linkage lie within the range of conformations seen in solution (Fig. 4) and several exocyclic torsion angles correspond well with both free and enzyme-bound forms of streptomycin (Fig. 5). It is notable that the S1–R2 linkage conformations lie within regions predicted computationally to have the highest energies (Fig. 4), which may represent either a problem with the structure or (if genuine) may account for the low binding affinity. Streptomycin also binds to a range of bacterial resistance enzymes. Experimental observations have concluded that regardless of the type of resistance enzyme bound to, the conformations of the aminoglycoside rings are identical^51,52^ and are similar to those observed here in solution.

### 4.3 Comparison with free crystal structures

Assessment of the unbound solution structure presented here with the CSD shows that in 90.4% of the observed macrostates the torsion angles are not unusual (according to Mogul^38^), which indicates that the 12 conformations are highly consistent with crystal structure data across most of the torsions (see Table 4). The G3 amine group (*χ*_2(G3)_) has an unusual torsion (when compared to the CSD using Mogul) with a slightly eclipsing interaction between the *N*-methyl group and the hydrogen on C2. The likely presence of an intramolecular hydrogen bond between G3 HN21 and R2 O31/O32, which requires this group to adopt this orientation, is considered to be the cause of this. The *ϕ*_2,3_ angle in Family 2 was also flagged as unusual by Mogul. While there are many observed torsions within a few degrees of that present in the set of solution conformational macrostates (−81 ± 9°), the majority of the recorded observations are larger, between −110° and −120°. It is hypothesized that this conformer is induced by steric and hydrogen-bond interactions between G3 and S1, and is besides very similar to that seen in the streptomycin oxime small molecule crystal structure (see below). The set of conformational microstates includes a slightly larger number of unusual torsions, although the total percentage of unusual torsions (when compared to the CSD) is only a little changed (73.8%), despite the increased coverage of conformational space. The dynamic 3D-structure determined in this work is therefore consistent with small-molecule crystal experimental data, giving an independent validation of the new NMR-methodology used to determine it.

Although the free crystal structure of streptomycin itself has not been reported, the coordinates of the R2 C3′ oxime derivative are available. As expected, this structure is in excellent agreement with existing CSD data. The only torsion in the free solution dynamic 3D-structure that is flagged as unusual by Mogul is *ϕ*_2,3_, with an angle of −64°. While this torsion is quite different from the majority of observations (−110° to −120°), it is nevertheless within a few degrees of several observed torsions. Analysis of the crystal structure indicates that this conformer is probably induced by formation of an intramolecular hydrogen bond between G3 O4 (acceptor) and S1 N31 (donor), as concluded for the dynamic 3D-structure when unbound above.

An overlay of the coordinates of the streptomycin oxime crystal conformation with the less populated Family 2 linkage conformation (Fig. 3) shows remarkable similarity. The S1–R2 glycosidic linkage conformation is similar to the average position adopted when unbound and clearly lies within the ensemble of conformations naturally explored in solution. The R2–G3 linkage conformation is also very similar to the Family 2 linkage average position and lies within the ensemble of conformations seen when unbound. The ribose ring pucker conformation (123°; ∼^4^*T*_3_) is again adjacent to that seen when unbound (144 ± 9°; ^4^*E*) and all the exocyclic torsion angles are also found at average positions of solution macrostates or within the unbound librational range (Fig. 5).

Given the packing interactions, numerous interactions with water molecules and selenate ions present in the free crystal, it is remarkable that the crystal conformation is so very similar to one of the two main conformations naturally present in aqueous solution. However, since the crystal structure is likely to be a low energy conformation, and the conformations seen in aqueous solution are also low (free) energy, this is not necessarily surprising. The replacement of the *gem*-diol group with the oxime is sterically very feasible and would not be expected to perturb the conformational energy landscape of the R2–G3 linkage on steric grounds (compare Figs. 2A and 5). However, since the oxime is only able to form one hydrogen bond with G3 HN21 (to the oxime nitrogen) while the *gem*-diol can make two (to O31 and O32, see above), the balance between Family 1 andFamily 2 linkage conformations may be shifted (and may therefore have favored the crystallization of streptomycin oxime in the Family 2 conformation).

Comparison of the ensemble of structures calculated here with energy surfaces at the two glycosidic linkages derived from computational chemistry (Fig. 4) shows that all experimentally-determined conformers correspond to low energy regions. Furthermore, there are no low energy basins predicted by computational chemistry that are not identified by this NMR method, providing confidence that all of the major conformational states populated in solution can be identified without a computational chemistry force field.

### 4.4 Free ligand solution structures in medicinal chemistry

Based exclusively on experimental data, this high resolution dynamic 3D-structure of streptomycin in free solution provides a concrete example of conformational pre-organization for binding. While the multiplicity of interactions present in a single protein-ligand complex is an enthalpy/entropy compromise that is almost impossible to separate,^53^ the fact remains that high-affinity ligands are quite likely to have low entropic barriers to binding, i.e., an ability to move readily from the free solution set of conformations to the bound set of conformations will naturally improve the binding affinity. The energy required to conformationally restrict and organize a free ligand into its bound conformation is always unfavorable and, while this penalty can be paid for via other binding energies (e.g., hydrogen bonding, desolvation), where high-affinity interactions are observed it is intuitively clear that a low conformational energy barrier is often likely to be a significant contributing factor. Therefore, it would not be surprising if conformational pre-organization for binding, as seen here for streptomycin, were in fact commonly observed.

A second important point to note from this study is that the free crystal 3D-structure, the bioactive conformation and other bound conformations are likely to be present in the free solution ensemble. However, free ligand crystal structures, while more predictive than computational chemistry (performed in vacuum), may bear little resemblance to the bioactive conformation.^54^ Therefore, accurate free ligand free solution 3D-structures are likely a more reliable predictor of the bioactive conformation than a single free ligand crystal 3D-structure when protein X-ray co-crystallographic structures are not available.

There are two immediately apparent situations where accurate unbound NMR solution conformations can be used in medicinal chemistry: (1) where the bioactive conformation is not available and hence a precise spatially-defined pharmacophore model cannot be defined, and (2) in molecular design and optimization for estimating the barrier to adoption of the bioactive shape when a protein X-ray co-crystal is available. In either case, contextualization of unbound-ligand 3D-structures, structure-activity relationships and protein co-crystallography data go hand-in-hand and are synergistic.

In the first case, the ability to determine accurate unbound 3D-structures allows ligand-based design methods to be applied.^55^ These approaches are particularly important for membrane-bound proteins or for disrupting protein–protein interactions, where co-crystallography is often intractable. Assuming (for high-affinity ligands) that the bioactive conformation is abundant in the free solution ensemble, a method can be envisaged to identify the bioactive conformation (and build a 3D-pharmacophore model) even for flexible ligands in the absence of a protein co-crystal. First, a set of high-affinity ligands for the same target and binding site (ideally with different scaffolds) would be identified, their solution structures determined and an initial phamacophore model proposed from structure–activity data. Common 3D-shapes are identified by overlaying unbound structures using 3D-pharmacophore points. By performing this for several ligands, a restricted set of overlaying macrostates may be identifiable, which would be hypothesized to contain the presumed bioactive conformation. Previous approaches have focused on overlaying ligands with their conformation determined by computational chemistry, which have not been accurate enough to perform such a process, but with the advent of accurate NMR-derived ligand free structures this becomes a real possibility. Consensus 3D-pharmacophore overlays are useful for rationalizing 3D-structure-activity relationships and can also be used to perform hit identification, scaffold hopping or lead optimization by virtual screening of diverse or focused compound collections using pharmacophores.

In the second case, when the bioactive conformation is already known, solution 3D-structures of ligands have significant utility for investigating the contributions to the thermodynamics and kinetics of binding. Effective affinity optimization requires an accurate theoretical understanding of the free energy associated with binding, which is dependent on bonding (solvation and intermolecular interactions) and the conformations of the receptor–ligand assembly (before and after binding).^53^ In particular, comparison of unbound ligand ensembles with the bioactive conformation allows the free energy of binding to be partitioned into components that are dependent on conformation and those that result from bonding. This is essential for rationalizing complicated structure–activity relationships that result from the fact that bonding opposes motion and motion opposes bonding during optimization.^56^ Furthermore, it can also be used to support the design of conformationally-restricted molecules (e.g., via cyclization) that can be used to drive the free conformation to the presumed bioactive conformation and perhaps to understand extraneous conformations that could be the source of off-target effects. These approaches can be used during preclinical drug development in both hit-to-lead and lead optimization processes.

## 5 Conclusions

A new NMR method is presented that has the capability to determine accurately the (unbound) conformations of ligands and their range of dynamic motion in a physiologically-relevant solvent environment that is independent of computational chemistry. The method is applied to the aminoglycoside streptomycin and reveals that the molecule has 12 conformational macrostates that can be grouped into two major families. Comparison with crystallographic data affirms that the bioactive conformation in complex with the 30S ribosomal subunit is well represented in the most populated family of free solution structures. Our observation, based exclusively on experimental data, provides another example of a high-affinity ligand that is conformationally pre-organized for binding to its target. Therefore, accurate ligand structures may (in some circumstances) provide an alternative route to the bioactive conformation when it cannot be determined by other means. When the bioactive conformation is already established, accurate free ligand structures provide a unique context for interpretation of the thermodynamics and kinetics of binding.

## Figures and Tables

**Table 1:** Summary of χ^2^ fit for all structural restraints

Dataset	No. of restraints	Total χ^2^	χ^2^/restraint
*2D-NOESY* (*D_2_O*)			
NOEs	167	139.1	0.8
no-NOEs	54	19.4	0.4

*2D-NOESY* (*H_2_O*)			
NOEs	9	13.2	1.5
no-NOEs	39	6.1	0.2

*Residual dipolar couplings*			
6% Gel	15	6.9	0.5
4.5% Gel	20	5.3	0.3
Scalar couplings	5	4.8	1.0
Total	309	194.9	0.6

**Table 2:** Composition of NOE restraints

Classification	NOEs	noNOEs	All
*Intraresidue*			
S1	9	5	14
R2	19	0	19
G3	32	1	33
Total	60	6	66

*Interresidue*			
S1–R2	39	19	58
R2–G3	56	17	73
S1–G3	21	51	72
Total	116	87	203

**Table 3:** Torsion angle conformational preferences for streptomycin in aqueous solution

Torsion^a^	Mode^b^	Mean angle	Libration amplitude^c^	Occupancy^d^
*Glycosidic linkages:*				
*ϕ*_12_	1	−75 ± 7°	16 ± 7°	1.00 ± 0.05
*ψ*_12_	1	−134 ± 17°	22 ± 6°	1.00 ± 0.05
*ϕ*_23_	1^e^	−103 ± 6°	6 ± 1°	0.62 ± 0.16
	2^e^	−81 ± 9°	10 ± 5°	0.38 ± 0.16
*ψ*_23_	1^e^	103 ± 8°	12 ± 7°	0.62 ± 0.16
	2^e^	173 ± 14°	25 ± 11°	0.38 ± 0.16
*Exocyclic:*				
*χ*_1(S1)_	1	78 ± 5°	17 ± 9°	0.50 ± 0.05
	2	162 ± 5°	17 ± 9°	0.50 ± 0.05
*χ*_3(S1)_	1	128 ± 13°	27 ± 13°	1.00 ± 0.05
*χ*_3(R2)_	1	180 ± 5°	20 ± 11°	0.51 ± 0.04
	2	300 ± 5°	20 ± 11°	0.45 ± 0.06
	3	60 ± 5°	20 ± 11°	0.04 ± 0.03
*χ*_2(G3)_	1	257 ± 8°	18 ± 7°	1.00 ± 0.05
*χ*_5(G3)_	1^e^	315 ± 7°	25 ± 14°	0.62 ± 0.16
	2^e^	296 ± 27°	25 ± 14°	0.38 ± 0.16

a Degrees of freedom and their associated dihedral angles are as defined in Figure 1.

b Macrostates are classified as distinct if rotation of the bond between the two conformations passes through a van der Waals maximum.

c The libration amplitude corresponds to the range of angles from the average value that the bond typically adopts in solution.

d The occupancy of each conformation is expressed as a proportion of the total for that bond.

e The two conformations adopted by the *ϕ*_23_, *ψ*_23_ and *χ*_2(G6)_ torsions are mutually dependent.

**Table 4:** Mogul analysis of torsion angles in streptomycin 3D-structures

Structure	Mogul category^a^				
	No hits (%)	Not unusual (enough hits) (%)	Not unusual (few hits) (%)	Unusual (enough hits) (%)	Unusual (few hits) (%)
*Dynamic 3D-structure:*					
Idealised-4D	0	67.3	23.1	9.6	0
Ensemble-4D	0	57.7	16.1	19.2	7.0

*Steptomycin oxime selenate free crystal:*					
STOSEH10^b^	0	83.3	12.5	4.2	0

*Protein-bound crystal structures:*					
1FJG^c^	4.4	65.2	0	21.7	8.7

a Mogul^36^ uses heuristics to identify torsion types that are similar to those in a query molecule, providing a statistical overview of previously observed values. The heuristics find a representative set of similar features, taking into account the local context of the query atoms. Torsions are divided into five categories. The ‘unusual (enough hits)’ category is of most concern, indicating that the current torsion is not consistent with a significant number of previous observations.

b CSD.

c PDB entry codes.
