# Supplementary material for: Quantification of free ligand conformational preferences by NMR and their relationship to the bioactive conformation
Source: Bioorg Med Chem. 2013 Sep 1;21(17):4976–87. doi: 10.1016/j.bmc.2013.06.056 (PMC3744816; doi:10.1016/j.bmc.2013.06.056)
Supplement: Supplementary data 1 — Supplementary Figures and Tables. [file mmc1.pdf]

Supplementary information

Contents

Tables

Chemical shift assignments

1. ^{1}H-chemical shifts for streptomycin in H_2O at 5ºC 2
2. ^{13}C and ^{15}N-chemical shifts for streptomycin in H_2O at 5ºC 3
3. ^{1}H and ^{13}C-chemical shifts for the aldehyde form of streptomycin in H_2O at 5ºC 4
4. ^{1}H-chemical shifts for streptomycin in D_2O at 25ºC 5
5. ^{13}C and ^{15}N-chemical shifts for streptomycin in D_2O at 25ºC 6

Coupling constants

6. $^{1}J_{CH}$ coupling constants for streptomycin in D_2O at 25ºC 7
7. J_{HH} coupling constants for streptomycin in aqueous solution 8

Temperature dependency of chemical shifts

8. ^{1}H-chemical shifts and temperature coefficients 9

Goodness of fit of observed and predicted data

9. Conformational restraints derived from scalar couplings 10
10. Conformational restraints derived from 2D-NOESY (D_2O) spectrum 11
11. Conformational restraints derived from 2D-NOESY (H_2O) spectrum 18
12. Conformational restraints derived from residual dipolar couplings 20

Density analysis of structural restraints

13. Number of restraints involving each proton used in the 4D-structure determination 22

Figures

1. Temperature coefficient fits 23
2. [^{1}H,^{15}N]-HSQC spectrum 27
3. [^{1}H]-1D spectrum of solvent-exchangeable nuclei 28
4. Exploration of second order model parameters 29
5. Likely transient intramolecular hydrogen bonds 30
6. Streptose ring pucker parameters 31

Calculation of error correction for residual dipolar couplings 32

Streptomycin 4D-structure co-ordinates

1. Idealised 4D-structure see accompanying file ‘streptomycin_idealised4d.sdf’
2. Ensemble 4D-structure see accompanying file ‘streptomycin_ensemble4d.sdf’

Chemical shift assignments

Table S1. ^{1}H-chemical shifts for streptomycin in H_2O at 5ºC

Residue	Atom[a]	Chemical Shift (ppm)[b]
S1	H1	3.479
	HN1	7.676
	H11*	6.877
	H12*	6.877
	H2	3.560
	HO2	6.932
	H3	3.626
	HN3	7.810
	H311(*pro-Z*)	7.095
	H312(*pro-E*)	6.869
	H32*	6.877
	H4	3.592
	H5	3.554
	HO5	6.127
	H6	3.474
	HO6	6.618

Residue	Atom[a]	Chemical Shift (ppm)[b]
R2	H1	5.291
	H2	4.407
	HO3	*7.195*[c]
	H3'	5.053
	HO31	-[d]
	HO32	-
	H4	4.445
	H5*	1.243
G3	H1	5.598
	H2	3.313
	HN21 & HN22	*8.862 & 8.358*[e]
	H2'*	2.854
	H3	3.935
	HO3	-
	H4	3.513
	HO4	6.485
	H5	3.706
	H61 (*pro-R*)	3.919
	H62 (*pro-S*)	3.830
	HO6	-

[a] Refer to Figure 1 (see text) for atom nomenclature.

[b] All chemical shifts were determined at a concentration of 50mM and 278.2 K, pH 6.0 in 10% D_2O and referenced directly relative to internal d_6-DSS. Std. error: ^{1}H ± 0.001 ppm.

[c] Resonance observed for a hydroxyl proton with no COSY correlations and was therefore assigned to R2 HO3; resonance could correspond to R2 HO31 or HO32.

[d] Hydroxyl proton was not observed due to fast chemical exchange with solvent.

[e] Prochiral stereo-assignment not achieved.

* Chemical shifts labelled with a star indicate nuclei that are degenerate.

Table S2. ^{13}C and ^{15}N-chemical shifts for streptomycin in H_2O at 5ºC

Residue	Atom[a]	Chemical Shift (ppm)[b]
S1	C1	61.440
	N1	87.483
	C1’	160.923
	N11	73.555
	N12	73.555
	C2	73.409
	C3	60.897
	N3	86.974
	C3’	160.489
	N31	74.378
	N32	73.555
	C4	80.224
	C5	76.007
	C6	74.136

Residue	Atom[a]	Chemical Shift (ppm)[b]
R2	C1	108.745
	C2	86.600
	C3	85.008
	C3’	91.903
	C4	80.110
	C5	15.025
G3	C1	96.695
	C2	63.886
	N2	-[c]
	C2’	34.504
	C3	71.860
	C4	72.095
	C5	75.841
	C6	63.031

[a] Refer to Figure 1 (see text) for atom nomenclature.

[b] All chemical shifts were determined at a concentration of 50mM and 278.2 K, pH 6.0 in 10% D_2O and referenced indirectly relative to internal d_6-DSS. Std. error: ± 0.020 - 0.050 ppm.

[c] Value not determined.

* Chemical shifts labelled with a star indicate nuclei that are degenerate.

Table S3. ^{1}H and ^{13}C-chemical shifts for the aldehyde form of streptomycin in H_2O at 5ºC

Residue	Atom[a]	Chemical Shift (ppm)[b]
R2	H1	5.404
	H2	4.733
	H3’	9.717
	H5*	1.173
	C1	68.889
	C2	84.678
	C3	89.805
	C3’	204.727
	C4	80.490
	C5	14.482

[a] Refer to Figure 1 (see text) for atom nomenclature.

[b] All chemical shifts were determined at a concentration of 50mM and 278.2 K, pH 6.0 in 10% D_2O and referenced directly or indirectly relative to internall d_6-DSS. Std. errors: ^{1}H ± 0.001; ^{13}C ± 0.020 ppm.

* Chemical shifts labelled with a star indicate nuclei that are degenerate.

Coupling constants within the aldehyde group and diagnostic for it:

$^{1}J_{H3'\text{-}C3'}$ = 172 Hz

$^{2}J_{H3'\text{-}C3}$ = 27 Hz

Table S4. ^{1}H-chemical shifts for streptomycin in D_2O at 25°C

Residue	Atom[a]	Chemical Shift (ppm)[b]
S1	H1	3.472
	H2	3.569
	H3	3.625
	H4	3.595
	H5	3.561
	H6	3.475

Residue	Atom[a]	Chemical Shift (ppm)[b]
R2	H1	5.292
	H2	4.392
	H3’	5.052
	H4	4.432
	H5*	1.249
G3	H1	5.571
	H2	3.308
	H2’*	2.860
	H3	3.945
	H4	3.513
	H5	3.713
	H61 (*pro-R*)	3.919
	H62 (*pro-S*)	3.823

[a] Refer to Figure 1 (see text) for atom nomenclature.

[b] All chemical shifts were determined at a concentration of 50mM and 298.2 K, pH* 6.0 in 100% D_2O and referenced directly relative to internal d_6-DSS. Std. error: ^{1}H ± 0.001 ppm.

* Chemical shifts labelled with a star indicate nuclei that are degenerate.

Table S5. ^{13}C and ^{15}N-chemical shifts for streptomycin in D_2O at 25°C

Residue	Atom[a]	Chemical Shift (ppm)[b]
S1	C1	61.482
	N1	86.743
	C1’	-
	N11	-
	N12	-
	C2	73.396
	C3	60.899
	N3	86.276
	C3’	-
	N31	-
	N32	-
	C4	80.407
	C5	75.970
	C6	74.189

Residue	Atom[a]	Chemical Shift (ppm)[b]
R2	C1	108.631
	C2	86.899
	C3	85.008
	C3’	92.078
	C4	80.191
	C5	15.082
G3	C1	96.955
	C2	63.946
	N2	34.567
	C2’	34.728
	C3	71.883
	C4	72.167
	C5	75.553
	C6	63.081

[a] Refer to Figure 1 (see text) for atom nomenclature.

[b] All chemical shifts were determined at a concentration of 50mM and 298.2 K, pH* 6.0 in 100% D_2O and referenced indirectly relative to internal d_6-DSS. Std. error: ± 0.020 - 0.050 ppm.

[c] Value not determined.

* Chemical shifts labelled with a star indicate nuclei that are degenerate.

Coupling constants

Table S6. $^{1}J_{CH}$ coupling constants for streptomycin in D_2O at 25ºC

Residue	Coupling constant[a]	Value (Hz)
S1	$^{1}J_{C1\text{-}H1}$	141.1
	$^{1}J_{C2\text{-}H2}$	147.4
	$^{1}J_{C3\text{-}H3}$	141.4
	$^{1}J_{C4\text{-}H4}$	146.4
	$^{1}J_{C5\text{-}H5}$	143.6
	$^{1}J_{C6\text{-}H6}$	145.8
R2	$^{1}J_{C1\text{-}H1}$	178.0
	$^{1}J_{C2\text{-}H2}$	149.5
	$^{1}J_{C3'\text{-}H3'}$	161.7
	$^{1}J_{C4\text{-}H4}$	147.2
	$^{1}J_{C5\text{-}H5*}$	128.1
G3	$^{1}J_{C1\text{-}H1}$	175.3
	$^{1}J_{C2\text{-}H2}$	146.9
	$^{1}J_{C2'\text{-}H2'*}$	144.4
	$^{1}J_{C3\text{-}H3}$	146.4
	$^{1}J_{C4\text{-}H4}$	144.8
	$^{1}J_{C5\text{-}H5}$	146.2
	$^{1}J_{C6\text{-}H61}$	144.2
	$^{1}J_{C6\text{-}H62}$	144.1

[a] Refer to Figure 1 (see text) for atom nomenclature.

[b] All coupling constants shifts were determined by direct measurement from the acquisition dimension of a ^{13}C-HSQC spectrum recorded without broadband ^{13}C-decoupling during acquisition on a sample of 50mM streptomycin, pH* 6.0, 100% D_2O at 298.2 K,. Std. error: $^{1}J_{CH}$ ± 0.5 Hz.

* Chemical shifts labelled with a star indicate nuclei that are degenerate.

Table S7. J_{HH} coupling constants for streptomycin in aqueous solution

Residue	Coupling constant[a]	Value (Hz)[b]
S1	$^3J_{H1\text{-}H2}$	-[c]
	$^3J_{H1\text{-}HN1}$	6.4
	$^3J_{H1\text{-}H6}$	-[c]
	$^3J_{H2\text{-}H3}$	9.2
	$^3J_{H3\text{-}HN3}$	8.5
	$^3J_{H3\text{-}H4}$	10.2
	$^3J_{H4\text{-}H5}$	8.6
	$^3J_{H5\text{-}H6}$	-[c]
R2	$^3J_{H1\text{-}H2}$	3.0
	$^3J_{H4\text{-}H5*}$	6.4
G3	$^3J_{H1\text{-}H2}$	3.2
	$^3J_{H2\text{-}H3}$	11.2
	$^3J_{H3\text{-}H4}$	9.9
	$^3J_{H4\text{-}H5}$	9.9
	$^3J_{H5\text{-}H61}$	2.4
	$^3J_{H5\text{-}H62}$	5.2
	$^3J_{H61\text{-}H62}$	-12.3

[a] Refer to Figure 1 (see text) for atom nomenclature.

[b] All coupling constants shifts were determined by direct measurement from 1D spectra recorded on a sample of 50mM streptomycin, pH 6.0, 10% (v/v) D_2O at 278.2 K,. Std. errors: J_{HH} ± 0.1 - 0.4 Hz.

[c] Value not determined because of lineshape distortion by strong coupling.

* Chemical shifts labelled with a star indicate nuclei that are degenerate.

Temperature dependency of chemical shifts

Table S8. ^{1}H-chemical shifts and temperature coefficients

Residue	Atom[a]	Chemical Shift (ppm) at a temperature (°C) of:[b]							Temperature coefficient ppb/K[c]
		3.77	5.04	9.85	14.96	19.96	25.06	35.07	
S1	HN1	7.691	7.686	7.667	7.646	7.627	7.608	7.570	-3.9
	HO2	6.951	6.948	6.941	-[d]	-	-	-	-1.5
	HN3	7.826	7.821	7.800	7.778	7.758	7.737	7.697	-4.1
	H311	7.110	7.106	7.090	7.070	-	-	-	-3.6
	H312*/H32**	6.884	6.880	6.873	6.864	6.852	6.839	6.815	-2.2
	HO5	6.132	6.117	6.061	5.999	5.942	5.877	-	-11.9
	HO6	6.664	6.657	6.602	-	-	-	-	-10.6
R2	H1	5.288	5.287	5.289	5.289	5.291	5.291	5.294	0.2
	H2	4.401	4.400	4.398	4.395	4.393	4.390	4.386	-0.5
	HO3	7.211	7.197	7.167	-	-	-	-	-6.9
	H4	4.445	4.444	4.442	4.438	4.436	4.433	4.429	-0.5
	H5*	1.244	1.245	1.245	1.245	1.247	1.247	1.248	0.1
G3	H1	5.593	5.590	5.585	5.579	5.575	5.569	5.560	-1.0
	H2	3.308	3.308	3.307	3.305	3.304	3.303	3.301	-0.2
	HN21[e]	8.868	8.860	8.838	8.811	8.794	8.757	-	-5.0
	HN22[e]	8.365	8.357	8.333	8.303	8.279	8.255	-	-5.2
	H2*	2.854	2.854	2.857	2.859	2.862	2.864	2.869	0.5
	HO4	6.515	6.508	6.471	6.434	-	-	-	-7.3
	H5	3.701	3.701	3.705	3.707	3.711	3.713	3.717	0.5

[a] Refer to Figure 1 (see text) for atom nomenclature.

[b] All chemical shifts were determined at a concentration of 50mM and pH 6.0 in 10% D_2O and referenced directly relative to internal d_6-DSS. Std. error: ^{1}H ± 0.001 ppm.

[c] Std. error ± 0.1 ppb/K.

[d] Proton was no longer observed due to fast chemical exchange with solvent.

[e] Prochiral stereoassignment not achieved.

* Chemical shifts labelled with a star indicate nuclei that are degenerate.

Goodness of fit of observed and predicted data

Table S9. Conformational restraints derived from scalar couplings

Restraint	Karplus parameters[a]				Observed value (Hz)	Mean predicted value (Hz)	mean χ^2
	A	B	C	ψ			
$^3J_{S1,H1\text{-}HN1}$	9.4	-1.1	0.4	0	6.4±1.0[b]	6.5	0.84
$^3J_{S1,H3\text{-}HN3}$	9.4	-1.1	0.4	0	8.5±1.0	8.7	0.27
$^3J_{R2,H1\text{-}H2}$	5.4	-0.9	1	-10	3.0±1.0	3.2	0.04
$^3J_{G3,H5\text{-}H61}$	8.31	-0.99	1.37	0	2.4±1.0	3.8	2.5
$^3J_{G3,H5\text{-}H62}$	9.58	-0.96	0.74	15	5.2±1.0	5.6	1.2

[a]Karplus equation in the form $^3J = A\cos^2(\theta+\psi) + B\cos(\theta+\psi) + C$, with ψ in degrees.

[b]Error applied to observed value is that of predictive capability of the Karplus relation, rather than measurement error (see Methods).

Table S10. Conformational restraints derived from 2D-NOESY (D2O) spectrum

NOEs

Donor residue	Donor nuclei	Acceptor residue	Acceptor nuclei	Measured height (x10^5)	Mean predicted height (x10^5)	Mean χ^2
S1	H3	S1	H1 & H6[a]	1.10E+03±4.20E+02	8.70E+02	0.22
R2	H1	S1	H1 & H6[a]	1.20E+02±49	1.20E+02	0.063
R2	H2	S1	H1 & H6[a]	29±12	16	1.3
R2	H4	S1	H1 & H6[a]	35±14	25	0.65
R2	H5*	S1	H1 & H6[a]	33±13	20	1.2
G3	H1	S1	H1	8.2±12	3.2	0.19
G3	H61	S1	H1	25±10	18	0.9
R2	H1	S1	H2	2.00E+02±80	97	1.6
R2	H2	S1	H2	23±9.1	13	1.8
R2	H31	S1	H2	6.8±23	1.8	0.049
R2	H4	S1	H2	22±8.8	8.9	2.2
R2	H5*	S1	H2	18±7.2	6.2	2.8
G3	H1	S1	H2	15±16	8	0.23
G3	H61	S1	H2	23±9.1	27	0.51
G3	H62	S1	H2	21±8.5	12	1.1
S1	H1 & H6[a]	S1	H3	1.30E+03±5.30E+02	8.70E+02	0.76
S1	H3	S1	H3	1.00E+04±4.00E+03	8.90E+03	0.087
R2	H1	S1	H3	2.60E+02±1.10E+02	65	2.9
R2	H2	S1	H3	79±37	56	0.48
R2	H31	S1	H3	17±17	6.4	0.38
R2	H4	S1	H3	58±23	29	1.6
R2	H5*	S1	H3	44±25	10	1.8
G3	H1	S1	H3	25±19	31	0.12
G3	H2*	S1	H3	6.7±2.7	5.7	0.31
G3	H3 & H61[a]	S1	H3	40±16	29	0.86
G3	H62	S1	H3	44±25	11	1.8
S1	H1 & H6[a]	S1	H4	1.20E+03±4.70E+02	1.40E+03	0.21
S1	H4	S1	H4	9.20E+03±3.70E+03	1.30E+04	1.5
R2	H1	S1	H4	1.60E+03±6.50E+02	1.60E+03	0.052
R2	H2	S1	H4	1.20E+02±49	74	0.98
R2	H31	S1	H4	22±20	9.7	0.38
R2	H4	S1	H4	1.10E+02±43	65	1.4
R2	H5*	S1	H4	73±29	37	1.8

G3	H1	S1	H4	43±26	33	0.24
G3	H2	S1	H4	22±8.9	5.6	3.5
G3	H2*	S1	H4	15±6	6.2	2.2
G3	H3	S1	H4	68±27	85	1.2
G3	H5	S1	H4	1.30E+02±51	83	0.9
G3	H62	S1	H4	37±15	35	0.48
R2	H1	S1	H5	2.60E+02±1.00E+02	84	2.9
R2	H2	S1	H5	42±17	37	0.21
R2	H31	S1	H5	20±24	11	0.15
R2	H4	S1	H5	83±33	1.20E+02	2.2
R2	H5*	S1	H5	42±17	49	0.75
G3	H1	S1	H5	12±23	10	0.016
G3	H1	S1	H6	7.6±12	4.2	0.091
S1	H1 & H6[a]	R2	H1	1.60E+02±64	1.20E+02	0.43
S1	H2 & H5[a]	R2	H1	4.70E+02±1.90E+02	1.80E+02	2.4
S1	H4	R2	H1	1.40E+03±5.50E+02	1.60E+03	0.14
R2	H1	R2	H1	1.60E+04±6.40E+03	1.60E+04	0.02
R2	H31	R2	H1	66±27	40	0.94
R2	H4	R2	H1	1.70E+02±69	1.40E+02	0.23
R2	H5*	R2	H1	1.40E+02±57	86	0.96
G3	H1	R2	H1	1.80E+02±72	1.60E+02	0.24
G3	H2	R2	H1	35±16	23	0.58
G3	H2*	R2	H1	60±24	28	2.2
G3	H3	R2	H1	2.10E+02±84	2.10E+02	0.12
G3	H5	R2	H1	8.50E+02±3.40E+02	7.30E+02	0.32
G3	H62	R2	H1	1.10E+02±44	80	0.53
S1	H1 & H6[a]	R2	H2	27±11	16	1.1
S1	H2 & H5[a]	R2	H2	68±27	50	0.47
S1	H3	R2	H2	61±25	56	0.28
S1	H4	R2	H2	1.20E+02±48	74	0.86
R2	H2	R2	H2	1.70E+04±6.90E+03	1.30E+04	0.47
R2	H31	R2	H2	6.00E+02±2.40E+02	8.80E+02	1.6
R2	H5*	R2	H2	1.50E+02±58	1.40E+02	0.021
G3	H1	R2	H2	2.10E+03±8.40E+02	2.20E+03	0.096
G3	H2	R2	H2	1.40E+02±57	1.40E+02	0.045
G3	H2*	R2	H2	3.30E+02±1.30E+02	2.20E+02	0.79
G3	H3	R2	H2	1.10E+02±45	89	0.37

G3	H4	R2	H2	27±13	25	0.071
G3	H5	R2	H2	4.20E+02±1.70E+02	2.00E+02	2.1
G3	H62	R2	H2	60±25	23	2.4
S1	H2 & H5[a]	R2	H31	20±8	12	0.93
R2	H1	R2	H31	46±19	40	0.12
R2	H2	R2	H31	6.10E+02±2.40E+02	8.80E+02	1.5
R2	H31	R2	H31	1.70E+04±6.80E+03	1.80E+04	0.027
R2	H4	R2	H31	7.80E+02±3.10E+02	8.00E+02	0.087
R2	H5*	R2	H31	7.60E+02±3.00E+02	8.30E+02	0.12
G3	H1	R2	H31	3.20E+02±1.30E+02	2.90E+02	0.34
G3	H2	R2	H31	29±13	24	0.21
G3	H2*	R2	H31	1.00E+02±40	97	0.16
G3	H3	R2	H31	21±10	16	0.26
G3	H5	R2	H31	21±10	19	0.33
G3	H61	R2	H31	11±7.1	5	0.83
S1	H1 & H6[a]	R2	H4	35±14	25	0.67
S1	H2 & H5[a]	R2	H4	1.00E+02±40	1.30E+02	1.1
S1	H3	R2	H4	48±31	29	0.4
S1	H4	R2	H4	91±47	65	0.71
R2	H1	R2	H4	1.40E+02±63	1.40E+02	0.014
R2	H2	R2	H4	4.90E+02±2.00E+02	7.50E+02	1.7
R2	H31	R2	H4	7.50E+02±3.00E+02	8.00E+02	0.12
R2	H4	R2	H4	1.60E+04±6.30E+03	1.40E+04	0.057
G3	H1	R2	H4	74±40	96	0.36
G3	H2	R2	H4	12±4.8	7.6	0.84
G3	H2*	R2	H4	33±13	18	1.3
G3	H5	R2	H4	23±23	18	0.083
R2	H1	R2	H5*	70±30	86	0.3
R2	H2	R2	H5*	93±39	1.40E+02	1.7
R2	H31	R2	H5*	3.90E+02±1.60E+02	7.80E+02	5.9
G3	H1	R2	H5*	33±17	34	0.079
G3	H2*	R2	H5*	49±20	18	2.5
S1	H4	G3	H1	36±17	33	0.28
R2	H1	G3	H1	1.60E+02±63	1.60E+02	0.18
R2	H2	G3	H1	1.90E+03±7.50E+02	2.20E+03	0.29
R2	H31	G3	H1	2.90E+02±1.20E+02	2.90E+02	0.34
R2	H4	G3	H1	1.20E+02±49	96	0.25

R2	H5*	G3	H1	47±21	34	0.44
G3	H1	G3	H1	1.60E+04±6.60E+03	1.10E+04	0.6
G3	H2*	G3	H1	1.50E+03±5.80E+02	9.60E+02	0.76
G3	H3	G3	H1	1.10E+02±45	1.80E+02	2
G3	H4	G3	H1	1.10E+02±43	1.20E+02	0.19
G3	H5	G3	H1	1.50E+02±59	1.50E+02	0.019
G3	H62	G3	H1	43±19	32	0.41
R2	H1	G3	H2	37±22	23	0.38
R2	H2	G3	H2	1.50E+02±60	1.40E+02	0.046
R2	H31	G3	H2	30±20	24	0.12
R2	H4	G3	H2	14±17	7.6	0.14
R2	H5*	G3	H2	12±17	5.1	0.17
G3	H2	G3	H2	1.50E+04±6.00E+03	1.30E+04	0.16
G3	H4	G3	H2	8.90E+02±3.60E+02	1.30E+03	1.1
G3	H5	G3	H2	1.20E+02±50	1.10E+02	0.038
G3	H62	G3	H2	74±30	43	1.2
R2	H2	G3	H2*	1.80E+02±74	2.20E+02	0.56
R2	H31	G3	H2*	59±24	97	2.9
G3	H1	G3	H2*	9.50E+02±3.80E+02	9.60E+02	0.16
R2	H1	G3	H3	1.40E+02±60	98	0.65
R2	H2	G3	H3	71±33	57	0.2
R2	H31	G3	H3	12±4.9	16	0.85
R2	H5*	G3	H3	23±18	8.5	0.71
G3	H1	G3	H3	1.20E+02±52	1.30E+02	0.016
G3	H2*	G3	H3	1.50E+02±61	1.30E+02	0.2
G3	H3	G3	H3	1.20E+04±4.80E+03	1.80E+04	1.7
G3	H5	G3	H3	1.00E+03±4.20E+02	1.20E+03	0.2
R2	H1	G3	H4	45±26	25	0.57
R2	H2	G3	H4	29±11	25	0.16
R2	H31	G3	H4	7.5±17	4.9	0.025
R2	H5*	G3	H4	9.7±3.9	2.2	3.8
G3	H1	G3	H4	1.40E+02±59	1.20E+02	0.093
G3	H2	G3	H4	9.80E+02±3.90E+02	1.30E+03	0.5
G3	H2*	G3	H4	1.00E+02±40	1.40E+02	1.2
G3	H4	G3	H4	1.40E+04±5.80E+03	1.80E+04	0.35
G3	H62	G3	H4	8.00E+02±3.20E+02	3.50E+02	2.1
S1	H4	G3	H5	1.80E+02±73	83	1.9

R2	H1	G3	H5	1.10E+03±4.30E+02	7.30E+02	0.76
R2	H2	G3	H5	5.60E+02±2.30E+02	2.00E+02	2.8
R2	H31	G3	H5	33±29	19	0.27
R2	H5*	G3	H5	44±32	12	0.99
G3	H1	G3	H5	2.20E+02±93	1.50E+02	0.67
G3	H2	G3	H5	1.70E+02±74	1.10E+02	0.76
G3	H2*	G3	H5	75±30	35	1.8
G3	H5	G3	H5	1.90E+04±7.50E+03	1.30E+04	0.68
R2	H1	G3	H61	1.10E+02±45	1.10E+02	0.17
R2	H2	G3	H61	76±33	32	1.9
R2	H31	G3	H61	8.1±14	5	0.077
R2	H5*	G3	H61	9.8±3.9	2.9	3.1
G3	H1	G3	H61	43±22	48	0.27
G3	H2	G3	H61	36±20	39	0.34
G3	H2*	G3	H61	21±8.5	14	0.92
G3	H4	G3	H61	3.50E+02±1.40E+02	1.70E+02	1.8
R2	H1	G3	H62	80±40	80	0.099
R2	H2	G3	H62	43±29	23	0.6
R2	H5*	G3	H62	9.2±23	2.5	0.087
G3	H1	G3	H62	33±26	32	0.036
G3	H2	G3	H62	55±31	43	0.21
G3	H2*	G3	H62	16±6.4	11	0.62
G3	H4	G3	H62	5.10E+02±2.00E+02	3.50E+02	0.79

[a]These restraints were included as overlaps between the NOE cross-peaks indicated.

noNOEs

Donor residue	Donor nuclei	Acceptor residue	Acceptor nuclei	Measured height (x10^5)	Mean predicted height (x10^5)	Mean χ^2
R2	H31	S1	H1	0±2.8	1.5	0.3
G3	H2	S1	H1	0±1.50E+02	0.65	1.90E-05
G3	H2*	S1	H1	0±13	0.95	0.0059
G3	H3	S1	H1	0±99	0.8	6.70E-05
G3	H5	S1	H1	0±57	2.5	0.002
G3	H2	S1	H2	0±6.3	1.8	0.087
G3	H2*	S1	H2	0±2.9	2	0.49
G3	H3	S1	H2	0±17	2.8	0.029
G3	H2	S1	H3	0±4.4	4.1	0.92
G3	H2	S1	H5	0±36	1.6	0.002
G3	H2*	S1	H5	0±2.9	2.6	0.84
G3	H3	S1	H5	0±29	2	0.0048
G3	H62	S1	H5	0±33	3	0.0083
R2	H31	S1	H6	0±2.8	2.2	0.63
G3	H2	S1	H6	0±58	0.99	0.00029
G3	H2*	S1	H6	0±3.3	1.2	0.14
G3	H3	S1	H6	0±40	1.9	0.0022
G3	H5	S1	H6	0±25	8.5	0.13
G3	H62	S1	H6	0±47	7	0.026
S1	H1	R2	H31	0±2.8	1.5	0.29
S1	H6	R2	H31	0±2.9	2.2	0.57
G3	H4	R2	H31	0±5.4	4.9	0.83
G3	H62	R2	H31	0±3.3	3.5	1.3
G3	H3	R2	H4	0±6.5	7.4	1.3
G3	H4	R2	H4	0±16	2.4	0.023
G3	H61	R2	H4	0±6.5	4.2	0.42
G3	H62	R2	H4	0±4.6	2.9	0.42
S1	H1	R2	H5*	0±11	5.6	0.27
G3	H2	R2	H5*	0±5.5	5.1	0.86
G3	H3	R2	H5*	0±11	8.5	0.57
G3	H4	R2	H5*	0±11	2.2	0.04
G3	H5	R2	H5*	0±15	12	0.67
G3	H61	R2	H5*	0±11	2.9	0.068
G3	H62	R2	H5*	0±4.3	2.5	0.35
S1	H1	G3	H1	0±25	3.2	0.016

S1	H6	G3	H1	0±18	4.2	0.053
S1	H2	G3	H2	0±97	1.8	0.00037
S1	H3	G3	H2	0±13	4.1	0.11
S1	H4	G3	H2	0±13	5.6	0.19
S1	H1	G3	H2*	0±19	0.95	0.0025
S1	H2	G3	H2*	0±19	2	0.012
S1	H3	G3	H2*	0±16	5.7	0.14
S1	H4	G3	H2*	0±19	6.2	0.12
S1	H5	G3	H2*	0±17	2.6	0.025
S1	H6	G3	H2*	0±14	1.2	0.0083
R2	H4	G3	H2*	0±26	18	0.51
G3	H62	G3	H2*	0±9.1	11	1.5
S1	H3	G3	H3	0±21	4.6	0.06
S1	H3	G3	H4	0±81	2.6	0.0011
R2	H4	G3	H4	0±5.7	2.4	0.18
S1	H3	G3	H61	0±26	25	1
R2	H4	G3	H61	0±9.3	4.2	0.21
R2	H31	G3	H62	0±2	3.5	3.3
R2	H4	G3	H62	0±4.8	2.9	0.37

Table S11. Conformational restraints derived from 2D-NOESY (H2O) spectrum

NOEs

Donor residue	Donor nuclei	Acceptor residue	Acceptor nuclei	Measured height (x10^5)	Mean predicted height (x10^5)	Mean χ^2
S1	H3 & H4[a]	S1	H311	5.4±2.2	2.1	2.5
R2	H1	S1	H311	0.63±0.46	0.16	1.1
R2	H2	S1	H311	1±0.62	1.1	1.5
G3	H1	S1	H311	1.2±0.68	1.4	0.94
G3	H3 & H61[a]	S1	H311	0.96±0.38	0.83	0.72
S1	H1 & H6[a]	S1	HN1	9±3.6	6.1	0.78
S1	H2 & H5[a]	S1	HN1	6.1±2.4	4.5	0.65
S1	H2 & H5[a]	S1	HN3	12±4.8	4.8	2.5
R2	H1	S1	HN3	3.7±1.9	3.2	2.5

[a]These restraints were included as overlaps between the NOE cross-peaks indicated.

noNOEs

Donor residue	Donor nuclei	Acceptor residue	Acceptor nuclei	Measured height (x10^5)	Mean predicted height (x10^5)	Mean χ^2
S1	H1	S1	H311	0±0.26	0.088	0.12
S1	H6	S1	H311	0±0.26	0.022	0.0077
R2	H31	S1	H311	0±0.16	0.068	0.27
R2	H4	S1	H311	0±0.3	0.095	0.17
R2	H5*	S1	H311	0±0.68	0.015	0.00064
G3	H2	S1	H311	0±0.43	0.11	0.07
G3	H2*	S1	H311	0±13	0.14	0.0002
G3	H4	S1	H311	0±0.36	0.043	0.015
G3	H62	S1	H311	0±0.27	0.24	0.96
R2	H31	S1	H312	0±0.13	0.039	0.15
R2	H5*	S1	H312	0±1.3	0.017	0.00025
G3	H2	S1	H312	0±0.39	0.024	0.0051
G3	H2*	S1	H312	0±0.58	0.031	0.005
S1	H3	S1	HN1	0±0.5	0.18	0.14
R2	H1	S1	HN1	0±0.39	0.028	0.0055
R2	H2	S1	HN1	0±0.52	0.0066	0.00017
R2	H31	S1	HN1	0±0.19	0.002	0.00012
R2	H4	S1	HN1	0±0.52	0.0095	0.00037
R2	H5*	S1	HN1	0±1.9	0.0072	1.50E-05
G3	H1	S1	HN1	0±0.52	0.0041	7.10E-05

G3	H2	S1	HN1	0±1	0.0014	2.10E-06
G3	H2*	S1	HN1	0±0.92	0.0014	2.40E-06
G3	H3	S1	HN1	0±0.59	0.002	1.20E-05
G3	H5	S1	HN1	0±0.6	0.0058	0.0001
G3	H61	S1	HN1	0±0.59	0.011	0.0004
G3	H62	S1	HN1	0±0.32	0.0052	0.00033
S1	H1	S1	HN3	0±1.8	0.18	0.012
S1	H6	S1	HN3	0±1.8	0.35	0.041
R2	H2	S1	HN3	0±1.1	0.25	0.053
R2	H31	S1	HN3	0±0.3	0.022	0.0053
R2	H4	S1	HN3	0±1.1	0.056	0.0026
R2	H5*	S1	HN3	0±5	0.025	2.70E-05
G3	H1	S1	HN3	0±0.8	0.44	0.36
G3	H2	S1	HN3	0±0.97	0.069	0.0053
G3	H2*	S1	HN3	0±2.1	0.038	0.00037
G3	H3	S1	HN3	0±1.1	0.1	0.011
G3	H5	S1	HN3	0±0.94	0.57	0.46
G3	H61	S1	HN3	0±1.1	1.7	2.6
G3	H62	S1	HN3	0±0.67	0.48	0.63

Table S12. Conformational restraints derived from residual dipolar couplings

Alignment with 4.5% polyacrylamide gel

First residue	First nucleus	First residue	First nucleus	Measured RDC (Hz)	Mean predicted RDC (Hz)	Mean χ^2
S1	C1	S1	H1	2.3±1	2.4	0.02
S1	C2	S1	H2	3.1±1.5	2.4	0.042
S1	C3	S1	H3	2.7±1	2.4	0.031
S1	C4	S1	H4	2.4±1	2.4	0.019
S1	C5	S1	H5	3.9±1.5	2.4	0.16
S1	C6	S1	H6	2.8±1	2.4	0.04
R2	C1	R2	H1	0.92±1	2	0.1
R2	C2	R2	H2	4.6±1	2.8	0.34
R2	C31	R2	H31	-0.51±1	0.5	0.32
R2	C4	R2	H4	2±1	1.6	0.15
G3	C1	G3	H1	0.67±1	1.6	0.2
G3	C2	G3	H2	0.02±1	0.5	0.046
G3	C3	G3	H3	0.84±1	0.52	0.03
G3	C4	G3	H4	1.1±1	0.47	0.072
G3	C6	G3	H61	-2±1	-1.7	0.13
G3	CN2	G3	H2*	-0.32±1	-0.97	0.079
R2	H4	R2	H5*	0.55±1	1.1	0.019
G3	H2	G3	H3	0.55±1	0.25	0.02
G3	H61	G3	H62	2.5±1	-0.2	3.5
G3	H5	G3	H62	0.48±1	0.19	0.021

Alignment with 6% polyacrylamide gel

First residue	First nucleus	First residue	First nucleus	Measured RDC (Hz)	Mean predicted RDC (Hz)	Mean χ^2
S1	C1	S1	H1	5.2±1.5	5.4	0.031
S1	C3	S1	H3	5.8±2	5.3	0.11
S1	C4	S1	H4	4.1±1	5.3	0.18
S1	C5	S1	H5	5.1±2	5.2	0.028
S1	C6	S1	H6	4.6±1.5	5.2	0.035
R2	C1	R2	H1	5.1±1	4.3	0.084
R2	C2	R2	H2	11±1	6.1	2.1
R2	C31	R2	H31	-1.2±1	1.1	1.7
R2	C4	R2	H4	3.7±1	3.4	0.083
G3	C1	G3	H1	4.3±1	3.4	0.33
G3	C2	G3	H2	2.2±1	1.1	0.27
G3	CN2	G3	H2*	-0.16±1	-2.2	0.62
G3	C3	G3	H3	-0.38±1	1.1	0.35
G3	C4	G3	H4	1.2±1	1	0.067
G3	C6	G3	H61	-3.1±1	-3.8	0.94

Density analysis of structural restraints

Table S13. Number of restraints involving each proton used in the 4D-structure determination

Residue	Atom[a,b]	No. of restraints
S1	H1	27
	HN1	16
	H2	25
	H3	22
	HN3	16
	H311(*pro-Z*)	14
	H312(*pro-E*)	4
	H4	22
	H5	13
	H6	26
R2	H1	34
	H2	36
	H3’	35
	H4	40
	H5*	32
G3	H1	36
	H2	34
	H2’*	34
	H3	27
	H4	22
	H5	25
	H61 (*pro-R*)	21
	H62 (*pro-S*)	29

[a] Refer to Figure 1 (see text) for atom nomenclature.

[b] Hydrogen atoms in streptomycin not included in this list did not have any structural restraints involving them.

* Chemical shifts labelled with a star indicate nuclei that are degenerate.

Figure S1. Temperature coefficient fits

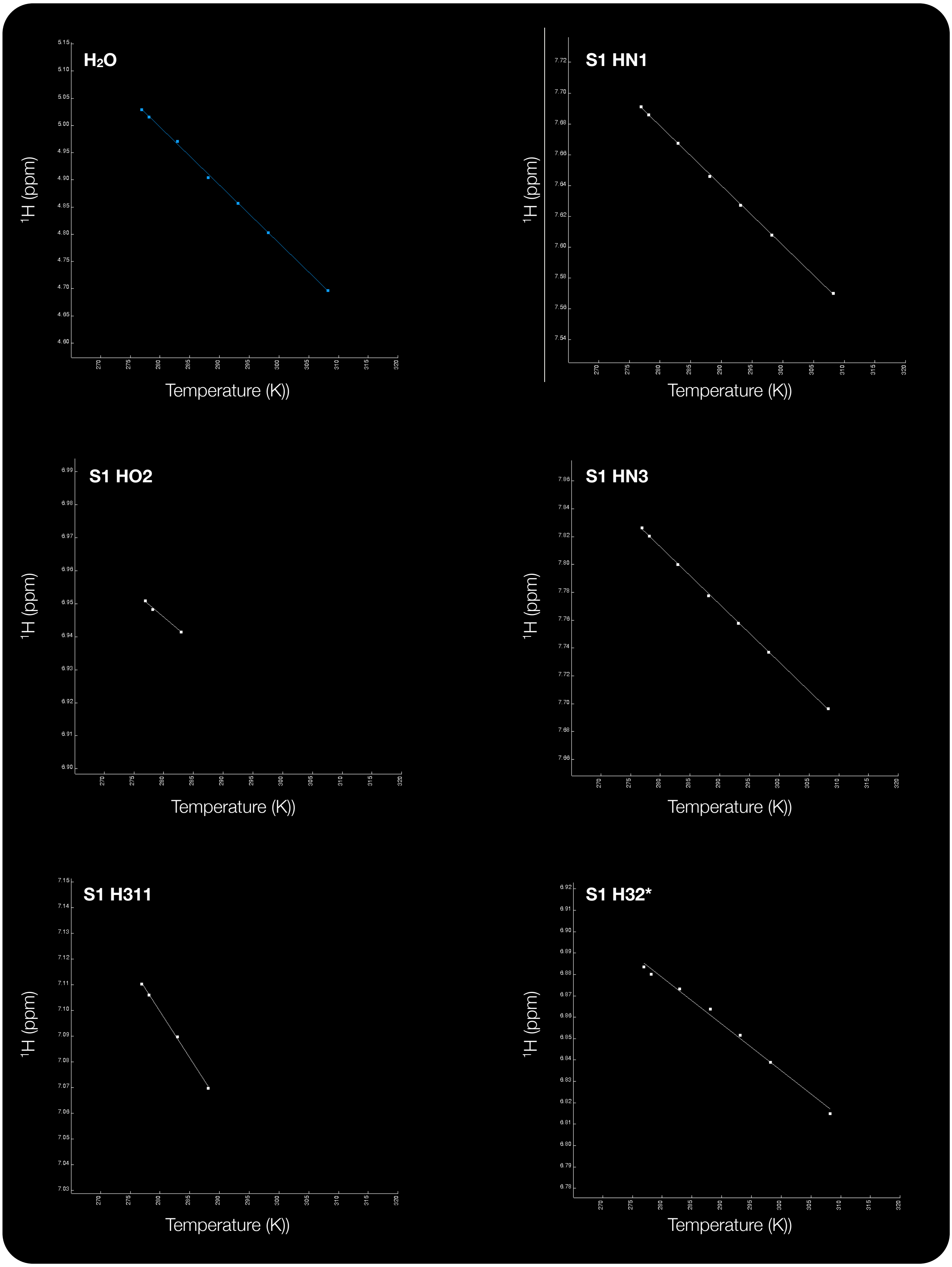

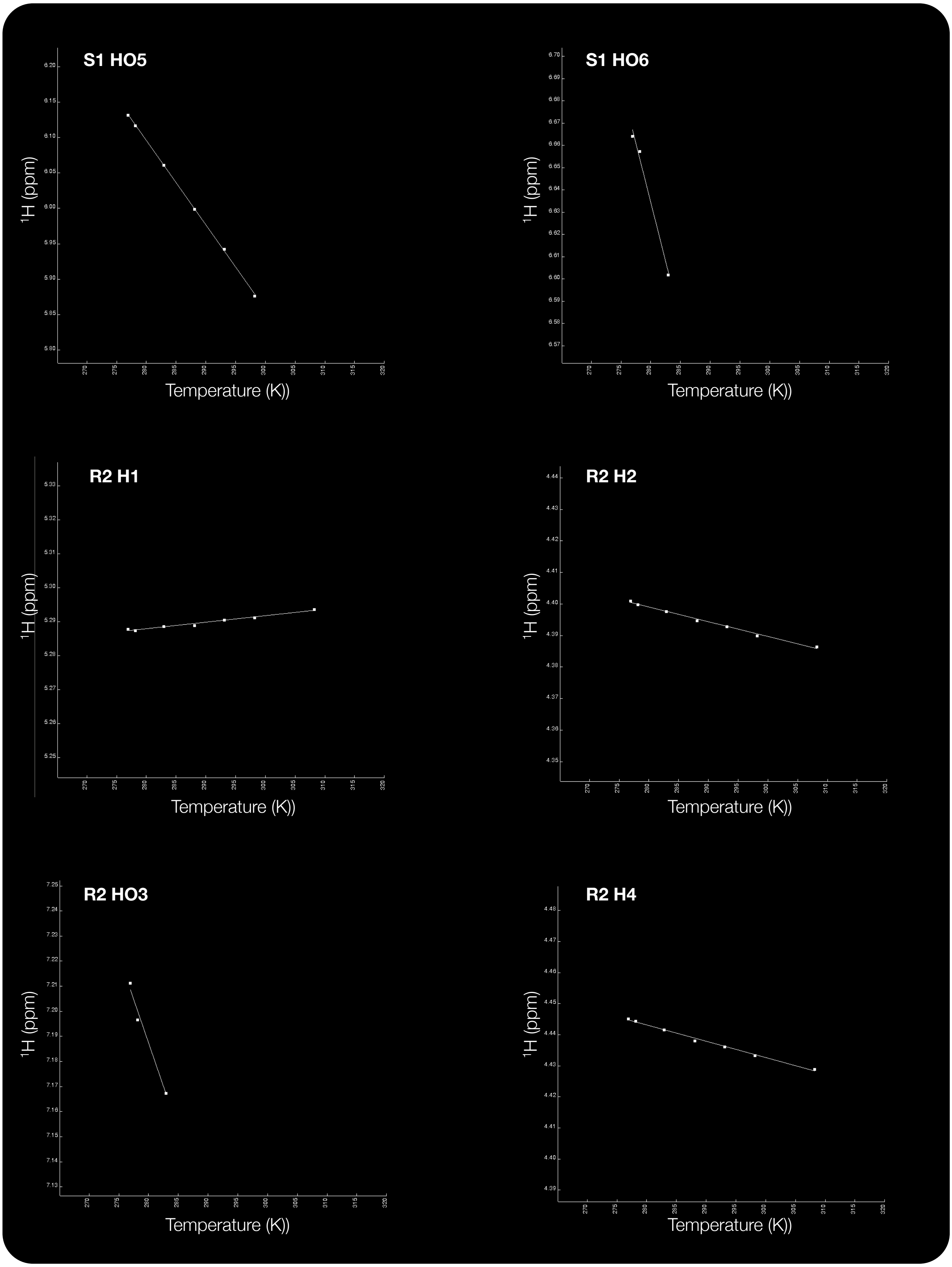
S1 HO5
1H (ppm)
Temperature (K))
S1 HO6
1H (ppm)
Temperature (K))
R2 H1
1H (ppm)
Temperature (K))
R2 H2
1H (ppm)
Temperature (K))
R2 HO3
1H (ppm)
Temperature (K))
R2 H4
1H (ppm)
Temperature (K))

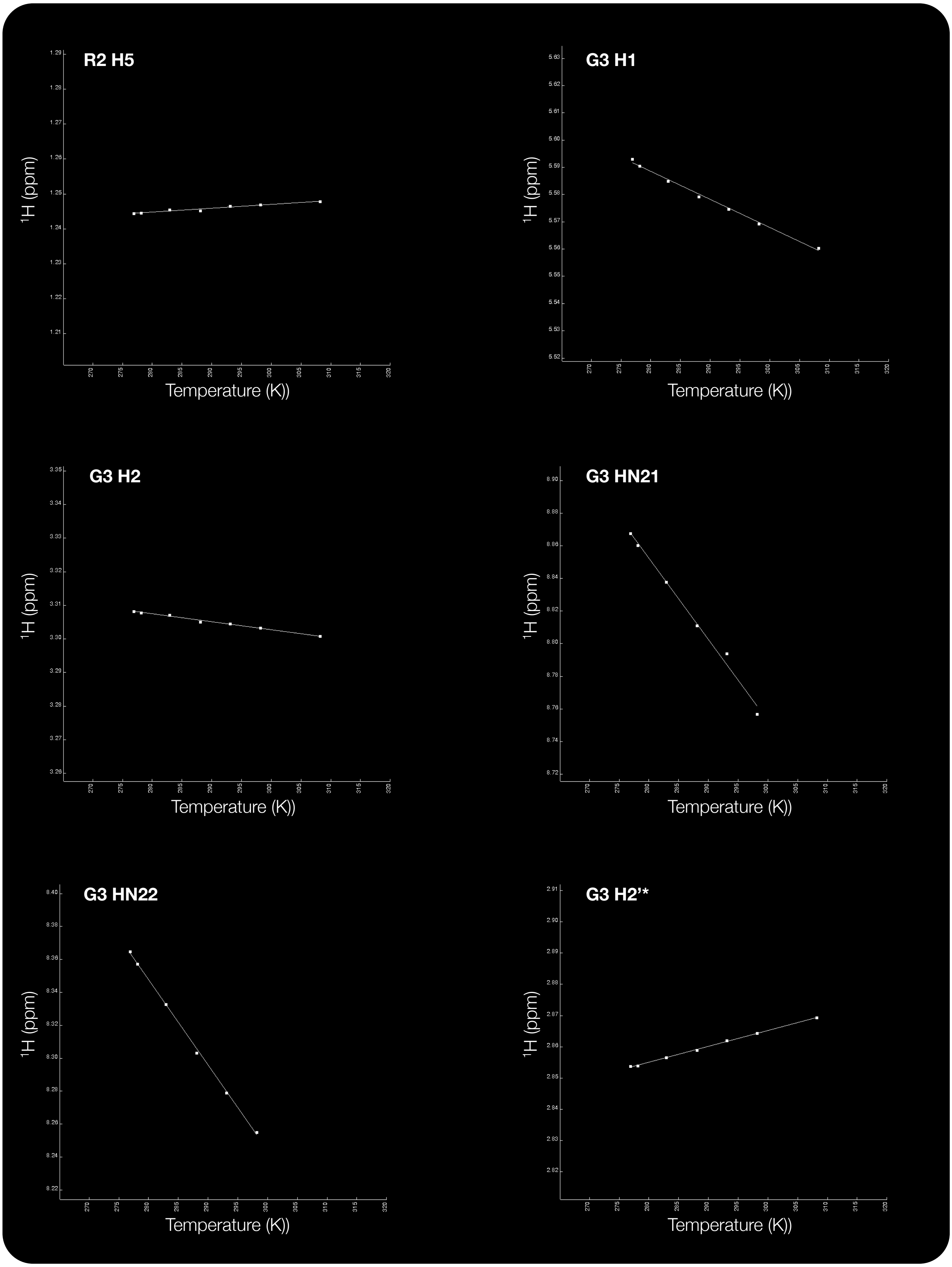
R2 H5
G3 H1
G3 H2
G3 HN21
G3 HN22
G3 H2'*
1H (ppm)
Temperature (K))

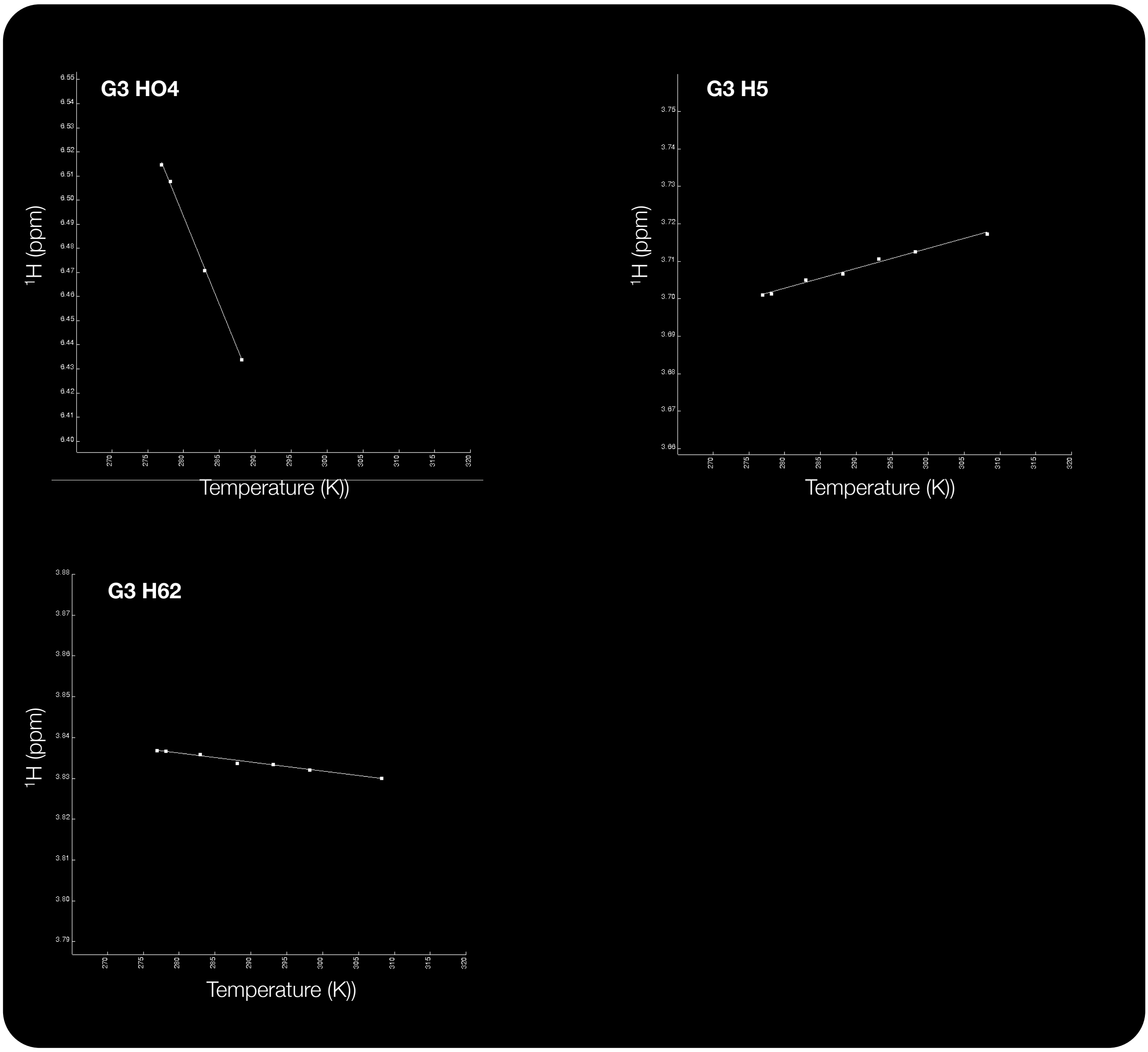
G3 HO4
1H (ppm)
Temperature (K))
G3 H5
1H (ppm)
Temperature (K))
G3 H62
1H (ppm)
Temperature (K))

Figure S2. [^{1}H,^{15}N]-HSQC spectrum

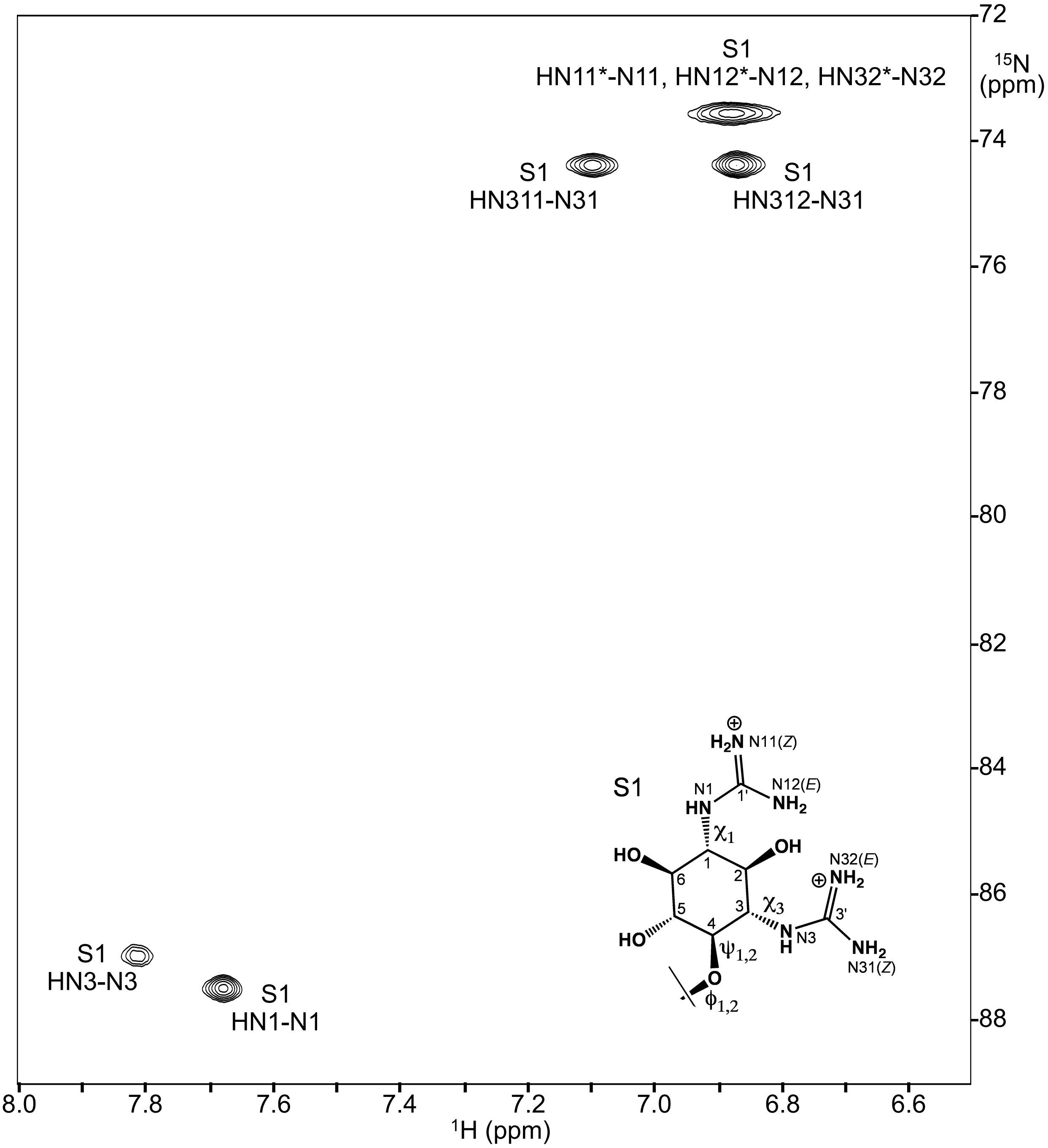

Figure S2. [^{1}H,^{15}N]-HSQC spectrum of streptomycin in H_2O at 5ºC (50mM, pH 6.0) showing resonance assignments for nuclei within the streptidine residue (S1) guanidinium sidechains. One of the four terminal amino groups is clearly in a unique environment. See inset for nomenclature.

Figure S3. [^{1}H]-1D spectrum of solvent-exchangeable nuclei

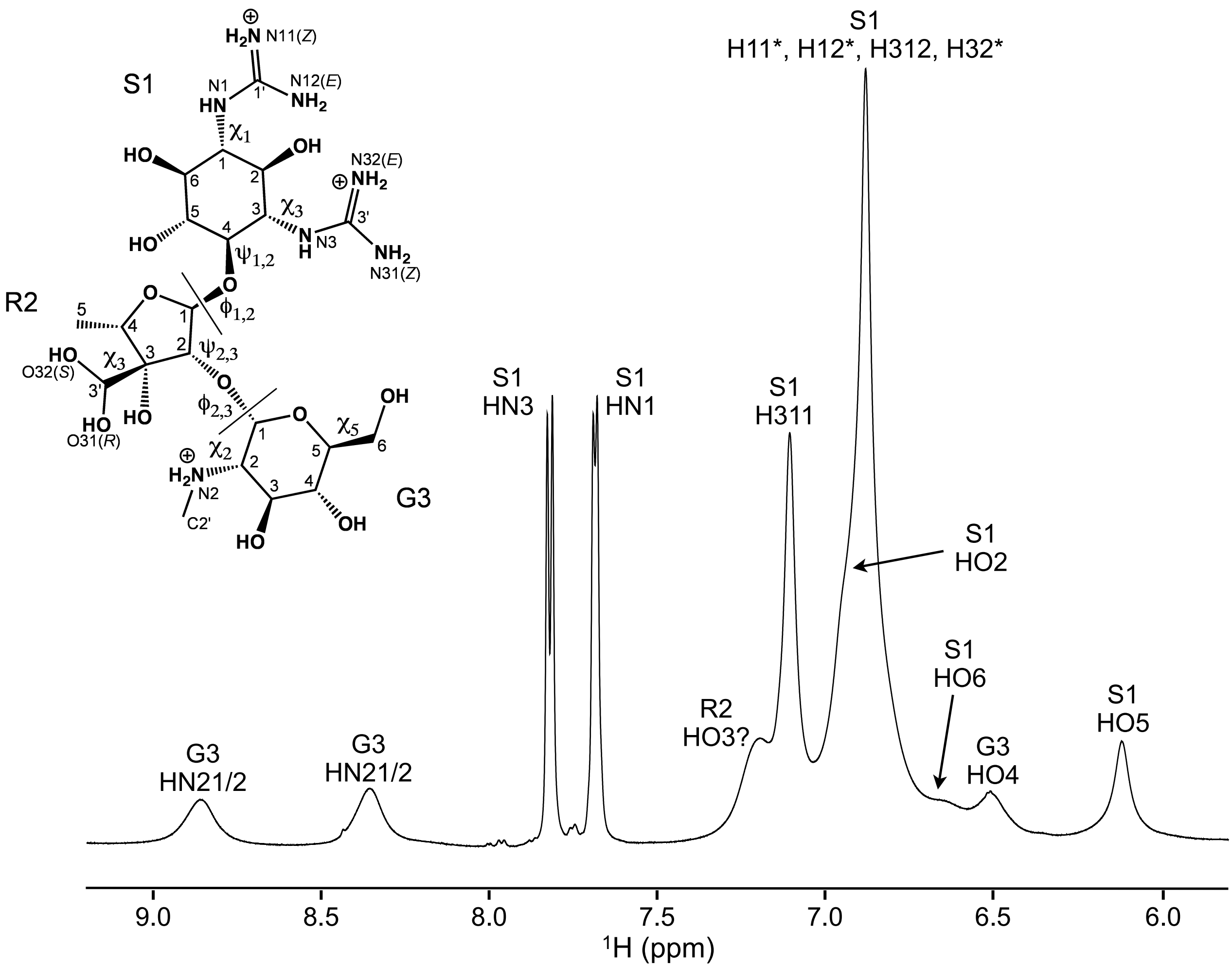

Figure S3. [^{1}H]-1D spectrum of streptomycin in H_2O at 5ºC (50mM, pH 6.0) showing resonance assignments for nuclei that can exchange with the solvent. See inset for nomenclature.

Figure S4. Exploration of second order model parameters

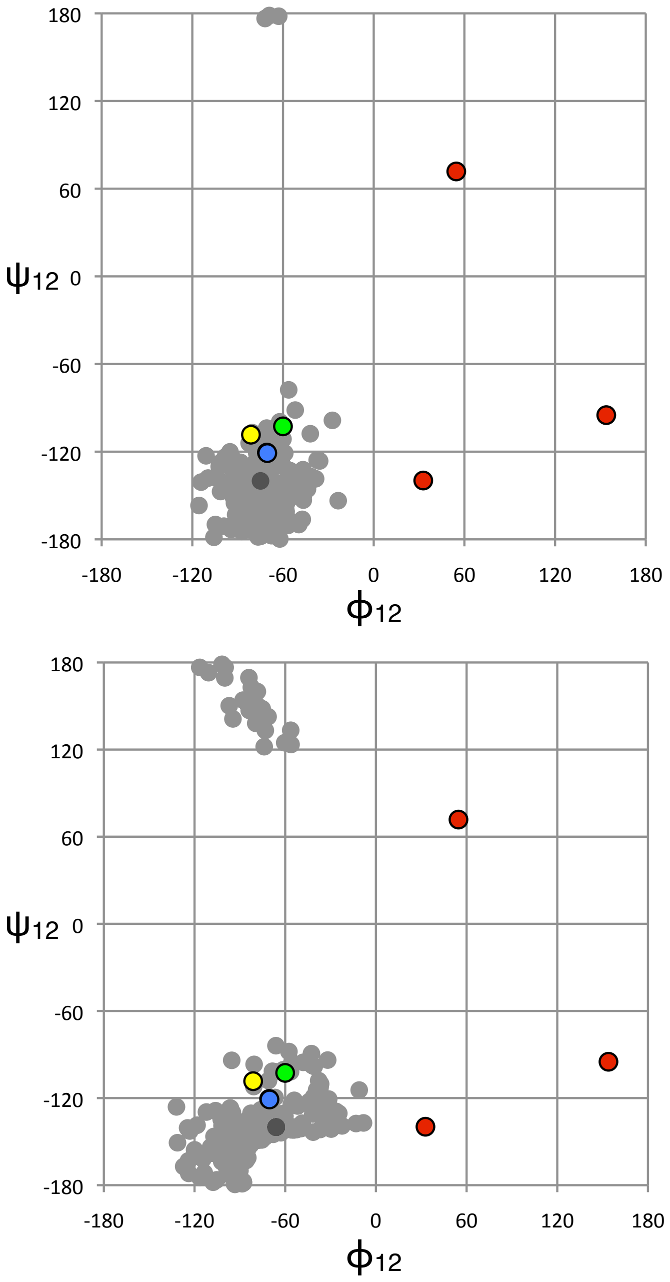

Figure S4. Comparison of the first order libration model (top) with a VDW steric limitation included for the second order of the libration model (bottom).

Figure S5. Likely transient intramolecular hydrogen bonds

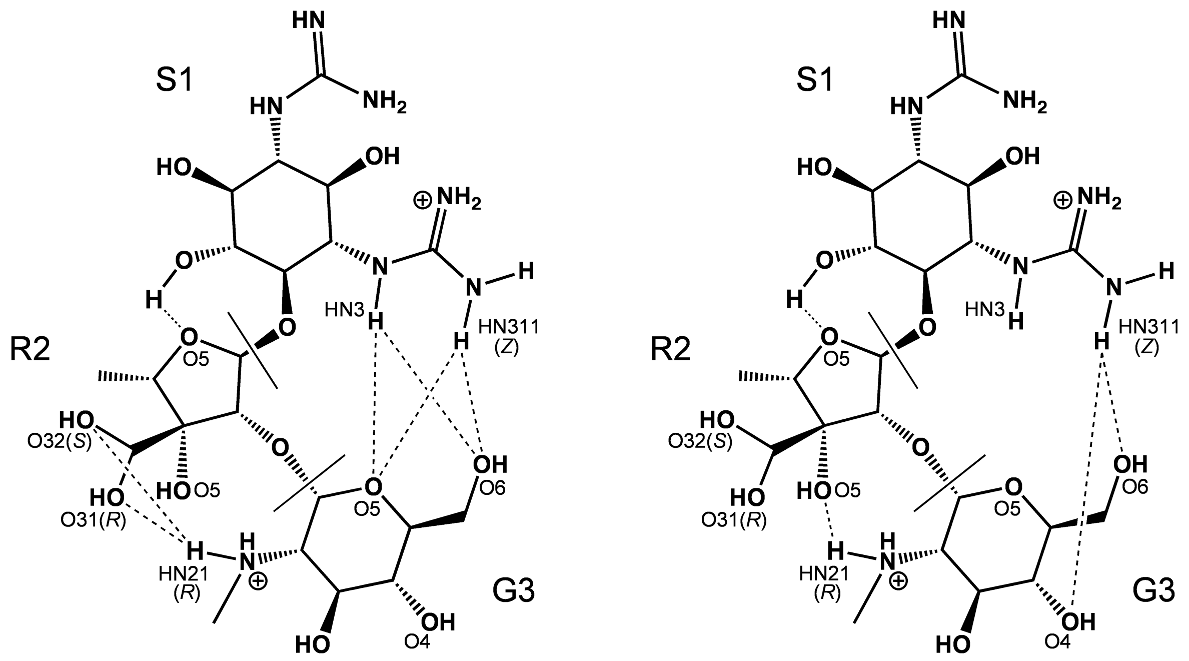

Figure S5. Likely intramolecular transient hydrogen bonds (dotted lines) present in streptomycin in aqueous solution. Potential hydrogen bonds in both the primary (left) and secondary (right) R2-S3 linkage two conformational families are shown.

Figure S6. Streptose ring pucker parameters

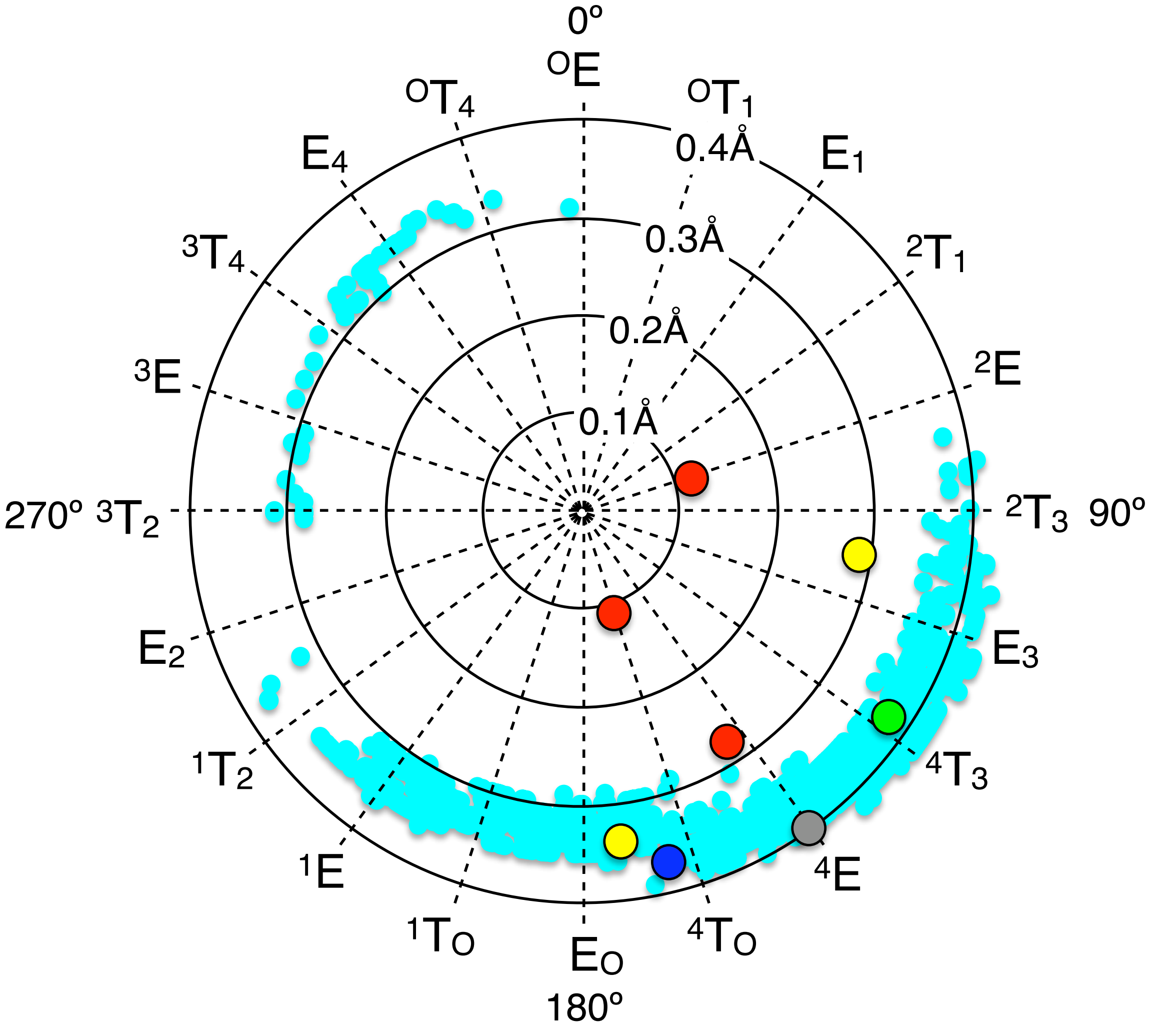

Figure S6. Comparison of the streptose ring (R2) pucker parameters (Kremer & Pople, JACS, 97, 1354-8) for the aqueous solution 4D-structure of streptomycin determined in this work (grey) with published crystal conformations (colours). Values from 1614 low-energy structures predicted using molecular dynamics simulation are shown in light blue. The distance from the centre of the circle represents the *q* value (Å) while the polar co-ordinate represents the ϕ angle (º). The ϕ angle corresponding to each canonical envelope and twist conformation is given. The bioactive conformation (*i.e.*, when streptomycin is bound to the ribosome, PDB code 1FJG) is shown in blue and the free crystal structure of the streptomycin oxime salt (Neidle *et al.,* 1978) is in green. The conformations of streptomycin bound to artificially-selected RNA aptamers (1NTA, 1NTB) are given in yellow and the three conformations measured from the off-target low affinity co-complex with aminoglycoside-6-adenyl-transferase are given in red (3HAV).

Calculation of error correction for residual dipolar couplings

For residual dipolar couplings (RDCs) the dependence on angle is highly non-linear and thus an extra error correction has to be applied. Correction of the error to take this into can be achieved by appling a scaling the error. The scaling (to produce an effective error ε_{exp}') can be derived in the following way. If θ is the angle between the major axis of alignment in the molecular frame, then starting from the equation defining residual dipolar couplings, equation (1) is obtained, which allows the calculation error to be obtained by differentiations, equation (2). Suitable approximations result in equation (3).

$$\mathrm{RDC} \propto \cos^2\theta - 1 \quad (1)$$

$$\text{Calculation error} = \left|\frac{d}{d\theta}(\cos^2\theta - 1)\right| \quad (2)$$

$$= |2\sin\theta\cos\theta|$$

$$= |\sin 2\theta|$$

$$\approx \frac{1}{2}(1 - \cos 4\theta) \quad (3)$$

Substituting the identity: $\cos 4\theta = 8\cos^4\theta - 8\cos^2\theta + 1$ into (3) and dividing this into the experimental error, results in equations (4) and (5), the latter of which is almost identical to equation (4), but avoids division by zero by having a minimum value of ¼ in the denominator and is therefore used in practice.

$$\Rightarrow \varepsilon_{exp}' \approx \frac{\varepsilon_{exp}}{4(\cos^2\theta - \cos^4\theta)} \quad (4)$$

$$\Rightarrow \varepsilon_{exp}' \approx \frac{\varepsilon_{exp}}{0.25 + 3(\cos^4\theta - \cos^2\theta)} \quad (5)$$

Using equation (5), it is possible to increase the total experimental error estimate (ε_{exp}) to take into account errors associated with predictions of residual dipolar couplings, which can then be used to more-accurately assess the degree of fit with the experimental data.
